# A method for tracking blue whales (*Balaenoptera musculus*) with a widely spaced network of ocean bottom seismometers

**DOI:** 10.1371/journal.pone.0260273

**Published:** 2021-12-15

**Authors:** William S. D. Wilcock, Rose S. Hilmo

**Affiliations:** School of Oceanography, University of Washington, Seattle, WA, United States of America; Pacific Northwest National Laboratory, UNITED STATES

## Abstract

Passive acoustic monitoring is an important tool for studying marine mammals. Ocean bottom seismometer networks provide data sets of opportunity for studying blue whales (*Balaenoptera musculus*) which vocalize extensively at seismic frequencies. We describe methods to localize calls and obtain tracks using the B call of northeast Pacific blue whale recorded by a large network of widely spaced ocean bottom seismometers off the coast of the Pacific Northwest. The first harmonic of the B call at ~15 Hz is detected using spectrogram cross-correlation. The seasonality of calls, inferred from a dataset of calls identified by an analyst, is used to estimate the probability that detections are true positives as a function of the strength of the detection. Because the spacing of seismometers reaches 70 km, faint detections with a significant probability of being false positives must be considered in multi-station localizations. Calls are located by maximizing a likelihood function which considers each strong detection in turn as the earliest arrival time and seeks to fit the times of detections that follow within a feasible time and distance window. An alternative procedure seeks solutions based on the detections that maximize their sum after weighting by detection strength and proximity. Both approaches lead to many spurious solutions that can mix detections from different B calls and include false detections including misidentified A calls. Tracks that are reliable can be obtained iteratively by assigning detections to localizations that are grouped in space and time, and requiring groups of at least 20 locations. Smooth paths are fit to tracks by including constraints that minimize changes in speed and direction while fitting the locations to their uncertainties or applying the double difference relocation method. The reliability of localizations for future experiments might be improved by increasing sampling rates and detecting harmonics of the B call.

## Introduction

Passive acoustic monitoring is an important tool for studying the spatial and temporal distribution, population density and habitat usage of vocalizing marine mammals, that complements visual surveys and tagging [1]. When acoustic sensors are deployed on the seafloor in suitably configured networks, they can be used to locate calls using multi-station methods [2]. For individuals that vocalize repeatedly, the resulting tracks provide additional constraints on animal behavior. Although passive acoustic monitoring is a relatively efficient method for long term observations, it is still costly to operate the large sensor networks required for monitoring and tracking over large areas. There are a limited number of capable Navy ranges that are optimized for tracking [3] but elsewhere in the oceans, data suitable for tracking can be hard to come by. For this reason, there is growing interest in taking advantage of ocean bottom seismometer (OBS) networks that are being increasingly deployed for earthquake monitoring along many continental margins and tectonic plate boundaries. Within the global academic community there are several hundred OBSs that are designed for deployments of about a year, and they are commonly deployed in networks of up to a few tens of sensors. Seismic networks generally sample at frequencies of only 50–200 Hz (historically, most often at the lower end of this range for long-term deployments) and so are only suitable for studying the baleen whale species that vocalize at low frequencies. OBSs with 50 Hz sampling rates are primarily sensitive to fin whales (*Balaenoptera physalus*) whose most common call is a 1-s 20 Hz chirp found throughout the oceans [4] and blue whales (*Balaenoptera musculus*) that have geographically distinct calls with the most common low-frequency calls generally centered between 15–25 Hz [5]. At sampling rates of 200 Hz, OBS have the potential to detect vocalizations from several other species including Bryde’s (*Balaenoptera brydei*) [6], Sei (*Balaenoptera borealis*) [7], Minke (*Balaenoptera acutorostrata)* [8] and Gray whales (*Eschrichtius robustus*) [9].

Several studies have obtained example fin whale tracks manually with local OBS networks using both time difference of arrival with multipath spacing [10] and time of arrival [11, 12]. Wilcock [13] developed a semi-automated method that uses a grid search method to model the times of multipath arrivals. It was used to obtain a large number of whale tracks over one year at a site on the Juan de Fuca Ridge [14]. Fin whale tracks can also be obtained from single stations using the three-component particle motions of the direct arrival [11, 15] and potentially from the spacing of multipaths in regions of complex bathymetry [16].

For blue whales, the call duration is often too long to identify multipaths or measure particle motions for the direct arrival and so multi-station networks are necessary for tracking. A number of studies have utilized seafloor seismometer or hydrophone networks to successfully track blue whales using time of arrival [17], time difference of arrival [18–20] and time difference of arrival combined with modeled amplitudes [10, 21]. In all of these studies, the calls were identified manually and only a few tracks were obtained. In all but one, the typical spacing of instruments was <15 km which simplified the task of associating the same call between stations. The one exception was a study off the Western Antarctic Peninsula where low ambient noise levels and a low density of calling animals, enabled tracks for Antarctic blue whales to be obtained with 3 stations spaced ~150 km apart after a careful search for matching calling sequences [19].

In this paper, we describe the development of a semi-automated algorithm to track Northeast Pacific blue whales using a large network of widely spaced ocean bottom seismometers in a setting where many calling animals can be present and noise levels are reasonably high because of shipping and climatology. A particular challenge addressed by this study is the need to consider very faint detections in order ensure that there are enough reporting stations to locate calls. This results in a data set with a lot of detections with a significant probability of being a false positive that can contribute to spurious localizations.

## Materials and methods

### Seismic data

**Fig 1:**
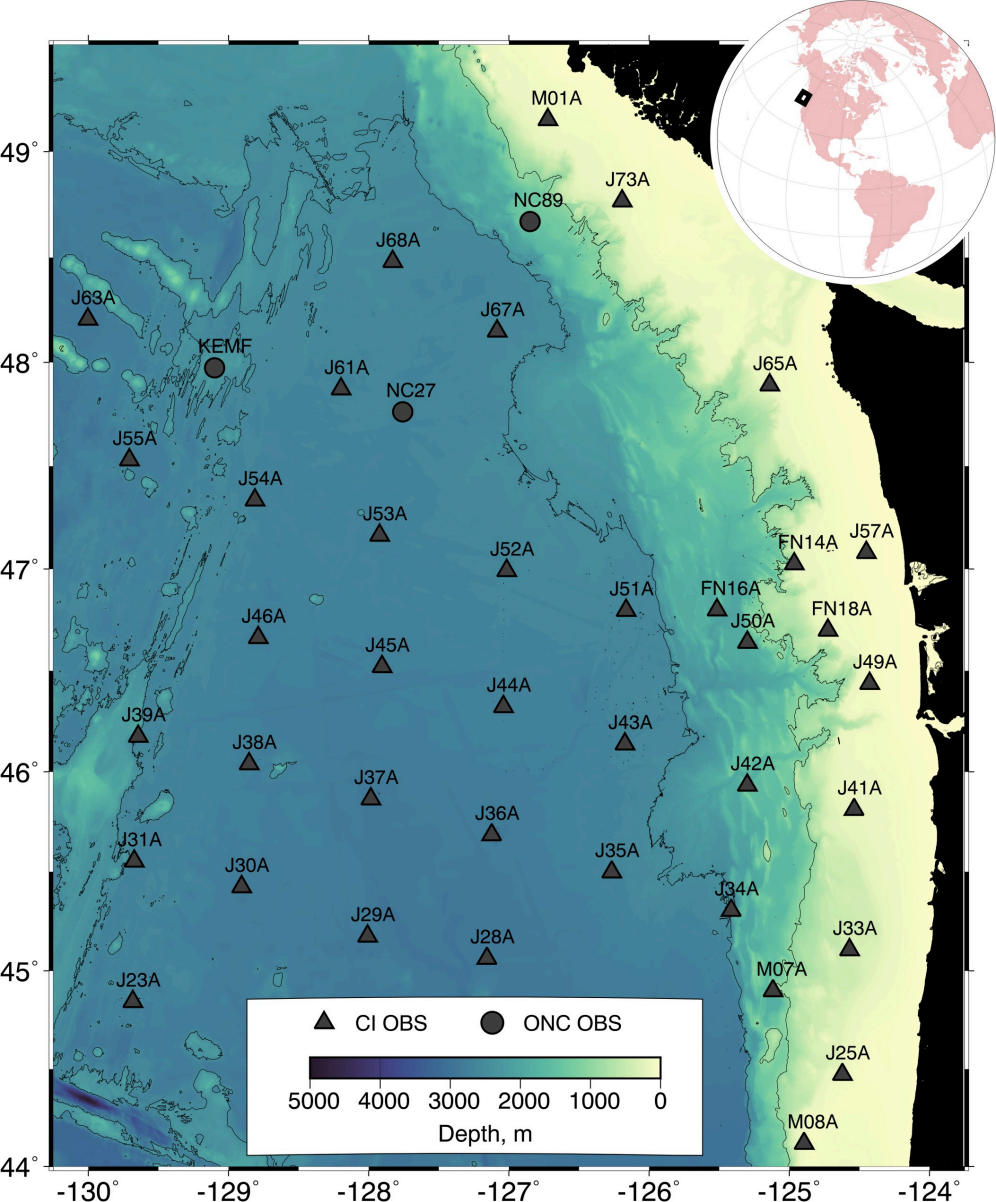
Seismic network. A bathymetric map of the continental margin and Cascadia Basin offshore the Pacific Northwest showing the configuration of the seismic network used to track blue whales. A total of 38 ocean bottom seismometers (OBSs) from the first year of the Cascadia Initiative (CI) experiment (triangles) were combined with three stations from the Ocean Networks Canada (ONC) NEPTUNE cabled observatory (circles). The full network was in operation from November 29, 2011, to May 13, 2012. Depths are shown by a color scale with contours at 200 m and 2500 m. The inset figure shows the location of the experiment on the globe. Bathymetric data were obtained from the Global Multi-Resolution Topography (GMRT) Synthesis (data DOI: 10.1594/IEDA.100001) [24] and are available for use under a CC BY 4.0 license. The figure was created with the Generic Mapping Tools software [25].

The seismic data used to track blue whales comes from a network of OBSs deployed as part of the Cascadia Initiative experiment, supplemented by seismometers on the Ocean Networks Canada (ONC) NEPTUNE cabled observatory (Fig 1). The Cascadia Initiative was an amphibious experiment designed to monitor seismicity and image earth structure on the Cascadia subduction zone and Juan de Fuca tectonic plate [22]. As part of this experiment, ~70 OBSs were deployed from late summer or early fall to summer of the following year over four years from 2011–2015. The experiment footprint alternated annually between a northern region off northern Oregon, Washington and southern British Columbia, and a southern region off northernmost California and Oregon. The network extended several hundred kilometers offshore with instrument spacings of ~70 km in deep water and ~35 km on the continental slope and shelf, except for two regions of concentrated deployments off Grays Harbor, Washington, and Cape Mendocino, California. Each instrument recorded the output of a Nanometrics Trillium Compact or Guralp CMG-3T seismometer that measured the three-component velocity of the seafloor and a pressure gauge that was primarily sensitive to frequencies below ~10 Hz. The majority of the OBSs sampled at 50 Hz with the rest sampling at 125 Hz. At the time of the experiment, the ONC NEPTUNE cabled observatory operated buried Guralp CMG-1T seismometers at several site on the continental slope and abyssal plain off Vancouver Island and several closely spaced short period GeoSENSE BH-1 corehole seismometers [23] on the Endeavour segment of the Juan de Fuca Ridge. These instruments sampled at 100 Hz or 200 Hz.

Here we use data from the vertical seismometer channel for the first year of the Cascadia Initiative OBS deployments (Fig 1). A total of 64 OBSs were deployed in this year in the northern footprint. After excluding stations that did not return good data at the frequency of blue whales or which were isolated spatially from other sensors, 39 OBS were suitable for multi-station tracking. These data were augmented by records from three cabled seismometers deployed on the ONC NEPTUNE cabled observatory. The network extends from the continental shelf across the margin and the Cascadia Basin to the Juan de Fuca Ridge. For the development of the localization algorithm, we have focused on a three-day test period from December 13–15, 2011 that includes many blue whale calls.

### Call detection dataset

**Fig 2:**
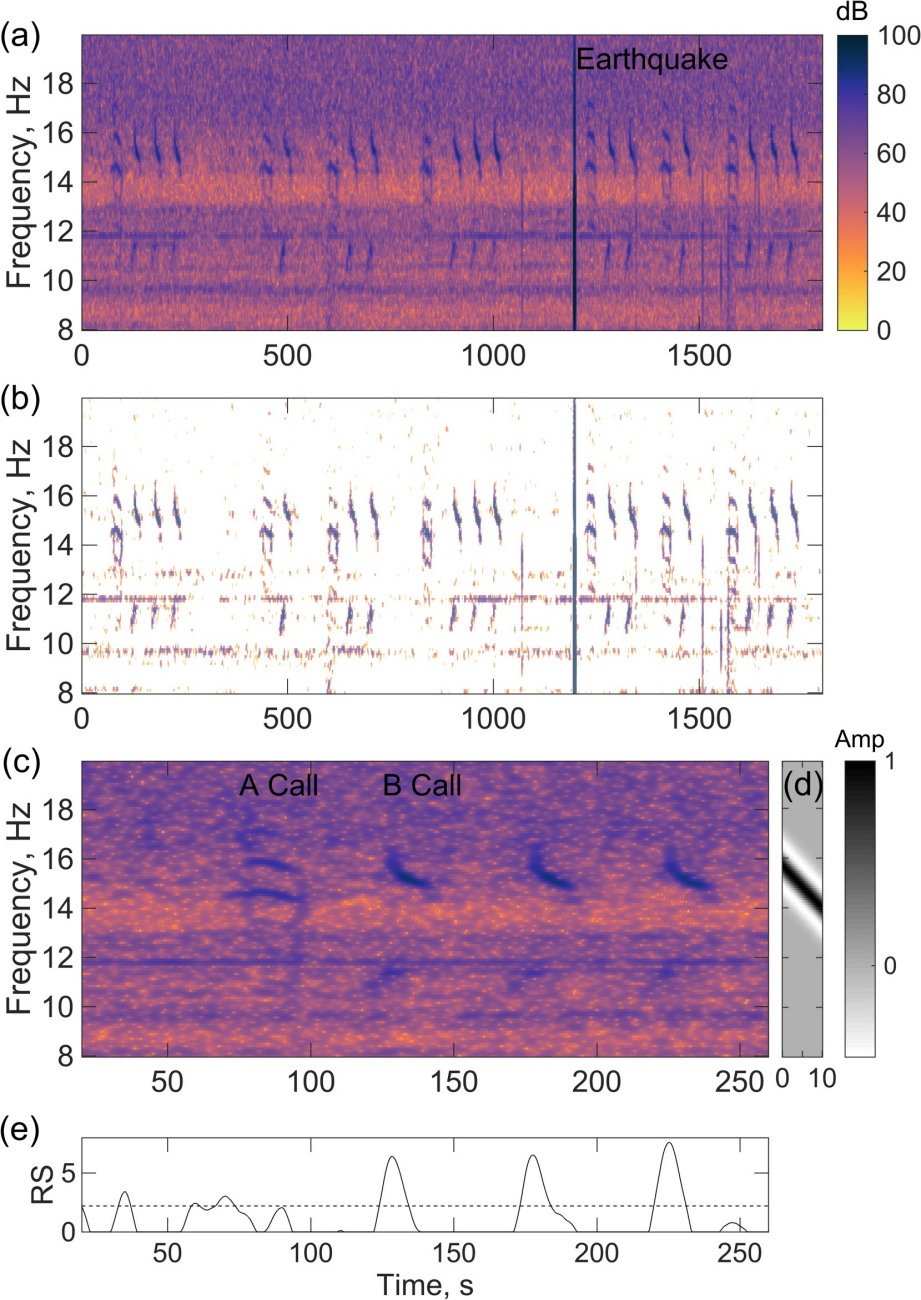
Example spectrogram and detections. (a) Spectrogram of 30 minutes of data with arbitrary scaling from station J53A starting at 03:30:30 UT on December 11, 2011, showing a sequence of strong Northeast Pacific blue whale calls. An earthquake is also apparent at ~1200 s as a vertical band of energy. (b) As for (a) but normalized by subtracting the median at each frequency and shown with a color scale for values that exceed the 90^th^ percentile value. (c) Four-minute segment of the spectrogram from (a) with A and B calls labeled. (d) Cross-correlation kernel that is tuned to the slope of the first half of the B call. (e) Recognition score (RS) for (c) with the minimum threshold of 2.2 shown as a dashed line. Three B calls are strong detections and both the A call and a faint feature early in the spectrogram register as weak detections.

The B calls of the Northeast Pacific blue whale were detected using the method of spectrogram cross-correlation whereby a detection kernel based on a template of the call is cross-cross correlated with a spectrogram of the data as a function of time with the frequencies matched [26, 27]. After normalizing the seismic records to a uniform sensitivity, continuous spectrograms were constructed for each station using a 2-s Hanning window with 80% overlap (Fig 2A–2C). Because the 50-Hz sampling frequency of the majority of the OBSs was insufficient to record the high-amplitude third harmonic of the B call and many of the instruments sampling at higher sampling rates were noisy at high frequencies [28], detections were based on just the first harmonic. The detection kernel (Fig 2D) was constructed from representative calls and comprises a single tone that sweeps down linearly from 15.7 Hz to 14.4 Hz over 10 s with a bandwidth of 0.5 Hz. Following Mellinger and Clark (2000), the detection kernel is implemented with the second derivative of a Gaussian function centered on the call frequency. This results in bands of negative values above and below the call frequency and ensures that the kernel sums to zero.

The kernel is cross correlated with the instrument spectrograms as a function of time with the frequencies matched. This leads to a time dependent recognition score which in our detection dataset is defined on a logarithm to base 10 scale. The scaling of the recognition score is arbitrary but the higher the value of the recognition score the more likely there is a B call. For our data set, a threshold of 2.2 was chosen as a conservatively low value. We identified as a detection all the peaks in the recognition score that exceeded this threshold and were separated from higher peaks by at least 10 seconds. For each detection, we recorded the time of the peak, the peak recognition score, and the duration for which the recognition score exceeded the threshold. The peak recognition score and duration are quite strongly correlated (correlation coefficient = 0.70).

**Fig 3:**
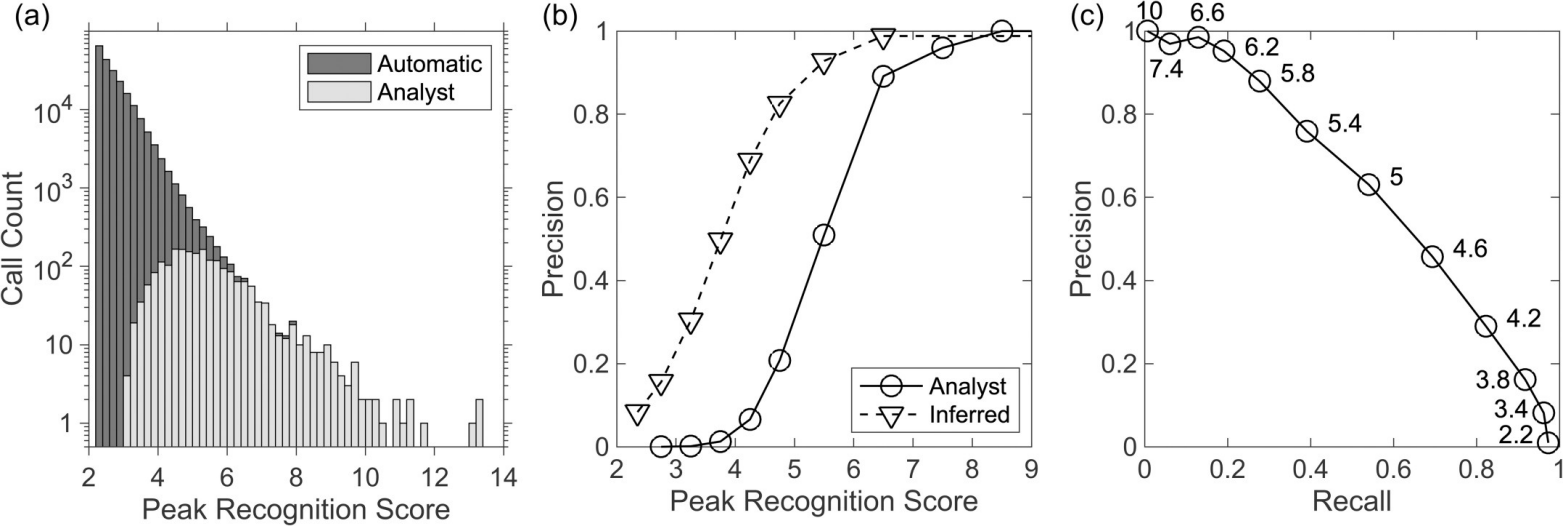
Performance of the detector. (a) Histogram with a logarithmic scale showing the call count for Cascadia Initiative station J28A for the automatic detector as a function of the peak recognition score (dark gray) and the calls that were identified independently by an analyst as a definite blue whale (light gray). The scaling of the peak recognition score is arbitrary. (b) Precision (number of true positives divided by the sum of true positive and false positives) of automatic blue whale detections as a function of the minimum peak recognition score determined by treating the analyst identifications as ground truth (solid line and circles) and as inferred by comparing detection rates between the calling and non-calling season for blue whales (dashed line and triangles). (c) Precision-recall curve for the automated detection based on treating the analyst detections as ground truth. The recall is the number of true positives divided by the sum of true positives and false negatives. Labels indicate the threshold for the peak recognition score.

To evaluate the detector, we compared on station J28A, the automated detections with a dataset of B calls that were confidently identified as a blue whale by a human analyst looking at the spectrogram independently (Fig 3). Because the automatic detection threshold was very low, all but 2.7% of the calls identified by the analyst registered as detections. Missed detections generally result from instances when the blue whale calls are overlapped by other transient signals, namely earthquakes, short duration events [28] and fin whale calls. Treating the analyst detections as ground truth, the precision of the automated detections is 0.98 and 0.91 for peak recognition scores >7 and in the range 6–7, respectively. However, these high threshold detections only account for 27% of the calls identified by the analyst and the peak recognition scores for some identified calls are as low as 3. Because the number of automated detections increases quickly with decreasing threshold (Fig 3B), the precision decreases rapidly at lower thresholds to 0.51 for peak recognition scores in the range 5–6 and 0.11 for the range 4–5.

**Fig 4:**
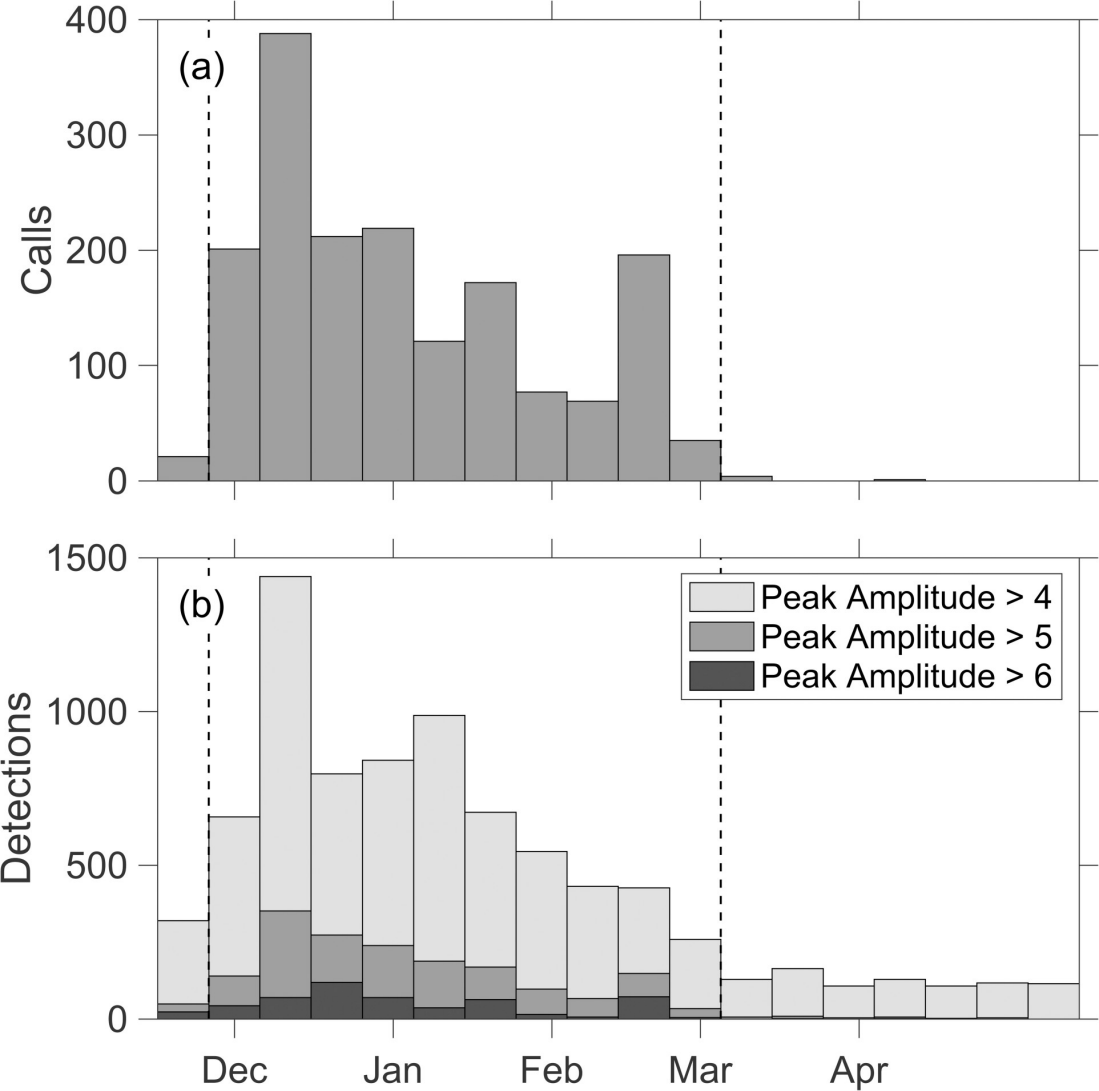
Histograms call times. A histogram showing call detections on station J28A as a function of time in 10-day bins. (a) Calls identified by an analyst as a definite blue whale. (b) Automated detections with peak recognition scores exceeding 4 (light gray), 5 (medium gray) and 6 (dark gray). Dashed vertical lines bound the assumed calling season.

A precision-recall curve (Fig 3C) illustrates the challenges of the detector. Achieving a high rate of recall requires a low precision leading to a large number of false detections. However, the analyst detections are based on an assessment that a feature in the spectrogram is definitely a blue whale and features that were possibly a blue whale where not included. It follows that many of the automated detections that were not identified by the analyst are likely true positives. An estimate of the precision as a function of the peak recognition score can be obtained after considering the distribution of analyst identified calls and detections with time (Fig 4). Blue whale calls are seasonal, and for OBS J28A all but 4 of the 1714 B calls identified by the analyst occur between November 25 and March 4. A comparison of the rates of detections in the calling and non-calling season for different ranges of peak recognition score shows that they are consistently higher in the calling season with the fractional difference decreasing as the peak recognition score decreases (Table 1). By making the simplifying assumptions that the rate of false detections is invariant throughout the year and that the blue whales do not call outside the identified calling season, the excess detections during the calling season can equated to the count of true positives. An estimate of the precision of the detector, *p*_r_, can be obtained according to

$$p_{r}(a)=\frac{\left(r_{c}(a)-r_{nc}(a)\right)}{r_{c}(a)}$$ (1)

where *a* is peak recognition score, and *r*_*c*_ and *r*_*nc*_, the rates of detections during the calling and non-calling seasons, respectively.

**Table 1:** Rate and estimated precision of automated detections.

Peak Recognition Score	Station J28A Detection Rate, day^-1^		Estimated Precision of Detections During Calling Season	
	Non-Calling Season	Calling Season	J28A	All Stations
2.2–2.5	457	498	0.08	0.07
2.5–3	370	438	0.16	0.16
3–3.5	144	207	0.30	0.38
3.5–4	47	94	0.50	0.62
4–4.5	13	43	0.69	0.79
4.5–5	3.7	21	0.82	0.88
5–6	1.2	16	0.93	0.95
>6	0.09	7.3	0.99	0.99

Data on station J28A are available from November 16, 2011, to May 15, 2012, and the calling season is defined as November 25 –March 4, the interval when all but 4 of 1714 confirmed detections were observed.

For OBS J28A (Table 1), the precision is only 0.16 for peak recognition scores in the range 2.5–3.0 but as the peak recognition score increase the precision increases rapidly (Fig 3B), reaching 0.5 and 0.82 for peak recognition score in the range 3.5–4 and 4.5–5, respectively. While the rate of true positives identified by the analyst is 17 per day during the calling season, the inferred rate of true positives with peak recognition scores exceeding 3.5 is 117 per day. Lowering this threshold to 2.5 doubles the inferred rate of true positives to 248 per day but at the expense of increasing the rate of false positives from 65 to 579 per day. To apply this approach to other stations, we inspected a sampling of automatic detections on many OBSs and concluded that detections with a peak recognition score ≥6, are consistently very likely true positives. For each OBS, we divided the data into 5-day bins and for each bin counted the number of automated detections within different intervals of peak recognition score. We then sorted the bins by the number of strong detections with peak recognition scores ≥6. We designated the smallest subset of bins that include 98% of the strong detections as occurring during the calling season and the remainder as occurring in the non-calling season. We then used Eq (1) to estimate the precision of the detector as a function of peak recognition score. The results are reasonably consistent between stations. Averaged over all stations (Table 1), the inferred precision increases from 0.07 for a peak recognition score of 2.2–2.5 to 0.95 and 0.99 for peak recognition scores of 5–6 and >6, respectively. While this analysis is reliant on the simplifying assumptions that there are no blue whale B calls outside the inferred calling season, and that the amplitudes of blue whale calls and the noise characteristics responsible for false detections are invariant throughout the year, it nevertheless provides a means to assess the general reliability of the detections incorporated into blue whale localizations.

### Localization algorithm

The call detection data set comprises a set of times that corresponds to peaks in the recognition score each with an estimated probability that the detection is a true positive. There is an extensive literature on marine mammal localization (see for example [29] for a brief review). Most approaches require a means to associate the same call on multiple stations before applying a separate localization step. Association is challenging for our dataset, not only because it potentially includes multiple blue whales making stereotyped calls at the same time, but also because our data set necessarily includes a large proportion of false positives because we must consider faint detections in order to ensure that calls are detected on enough stations for multi-station localization.

Nosal [29] describes a probabilistic method for tracking multiple marine mammals using time difference of arrival or time of arrival which is attractive for our dataset because it requires no a priori assumptions about which arrival belongs to which call. Using time of arrival, a likelihood function, *L*, for a single call recorded by a network of stations can be expressed as a product of the travel time misfits according to

$$L(x,O)=\prod_{i=1}^{N}exp[-\frac{1}{2\sigma_{i}^{2}}{\left(T_{i}-{\hat{t}}_{i}(x)-O\right)}^{2}]$$ (2)

where ***x*** and *O* are the position and origin time of the call, *i* the index of *N* stations recording the call, *T*_*i*_ the observed call arrival times, *σ*_*i*_ the arrival time uncertainties and ${\hat{t}}_{i}(x)$ the predicted travel times. For multiple calls, this expression is modified to

$$L(x,O)=\prod_{i=1}^{N}\underset{k}{\max}\left\{exp[-\frac{1}{2\sigma_{i,k}^{2}}{\left(T_{i,k}-{\hat{t}}_{i}(x)-O\right)}^{2}]\right\}$$ (3)

where *k* is the index of arrival times on a given station occurring after the call time, and the likelihood function is calculated by choosing the arrival time on each station that maximizes its value. A grid search in space and time can then be used to find maxima in *L* which will correspond to potential locations. When the arrival time data set includes only true arrival times with paths that match those used to model travel times and when arrival times are available for each call on each station, each maximum in *L* with a value close to unity will correspond to a call location. When spurious arrival times are present, the method is likely to lead to erroneous localizations. In the case of an animal calling repeatedly, post-processing can be used to identify those call locations consistent with a single animal track. Nosal [29] suggests that when more stations are present than necessary to locate the call unambiguously and when arrival times may not always be present on all stations for a given call, it may be desirable to maximize over several subsets of stations, each containing at least the minimum number of stations necessary to locate a call.

There are challenges to applying the method of Nosal [29] to the blue whale date set. First, the method is computationally demanding, and those demands increase with the size of the network and the duration of the observations. Second, spurious arrival times can lead to erroneous locations and as noted above, our dataset necessarily includes a large proportion of false positives. Third, for a large network, the approach of subsetting stations to account for stations that may not record a particular call adds to the computational burden and may act to increase the number of erroneous localizations from spurious detections by removing the redundant arrival times necessary to identify them.

To reduce the computational demands, we limit the search in space and time to solutions that are consistent with the time of a strong “master detection” that is the earliest arrival time in the solution. Each detection time with an estimated probability of being a true positive that exceeds a high threshold is designated in turn as a master detection and its time is combined with all detection times exceeding a lower probability threshold that follow within a feasible time window on stations at a feasible distance. A likelihood function, is constructed from these detection times according to

$$L(x)=\int_{-m\sigma_{1,1}}^{m\sigma_{1,1}}\prod_{i=1}^{N}max(\underset{k}{\max}\{exp[-\frac{1}{2\sigma_{i,k}^{2}}{\left(T_{i,k}-{\hat{t}}_{i}(x)-O(x)-t\prime \right)}^{2}]\},q_{small})dt\prime$$ (4)

where the sum is over the *N* stations with detections and *O(****x***) is the call origin time calculated at a given location based on the time of the master detection according to

$$O(x)=T_{1,1}-{\hat{t}}_{1}(x)$$ (5)

where the master detection time is assigned the indices *i* = 1 and *k* = 1. Since the precise call origin time is not of interest, the integral over *t’* in Eq (3) sums the likelihood function at a given location over all call origin times that are consistent with the master detection time to an acceptable multiplier, *m*, of its uncertainty, *σ*_1,1_. To prevents stations with no fitting detections from influencing the maximum of *L*, a uniform small value, *q*_*small*_, is substituted into product when the likelihood at a particular station is very low.

Maximizing Eq (3) will yield the solution that fits the master detection and as many other detections as possible, but in some instances, it can fail to yield the correct location if it fits low probability detections or detections on distant stations at the expense of higher probability detections nearer the call location. To overcome this, a second approach is considered which seeks to maximize the number of high probability detections on stations near the whale location. For the *i*^th^ station, we define a function *f*_*i*_ which is set to zero or unity depending on whether there is a fitting arrival time according to

$$\begin{array}{l} f_{i}(x,t^{\prime })=1\;\mathrm{if}\;\underset{k}{\min}|\frac{T_{i,k}-{\hat{t}}_{i}(x)-O(x)-t^{\prime }}{\sigma_{i,k}^{2}}|\leq m^{\prime }\\ f_{i}(x,t^{\prime })=0\;\mathrm{otherwise} \end{array}$$ (6)

where *m’* defines the maximum number of standard deviations for an acceptable detection time misfit. A solution that favors fitting arrival times for a high probability detection near the location is expressed as the maximization of

$$E(x,t^{\prime })=F(x,t\prime )\sum_{i=1}^{N}f_{i}(x,t\prime )\frac{p(i,k)}{\max\left(|x-x_{i}|,R\right)}$$ (7)

where *p*(*i*,*k*) is the estimated probability that the detection is a true positive, and max (|***x***−***x***_*i*_|, *R*) is the distance of the station at position ***x***_*i*_ from the call position subject to a minimum value, *R*. *R* prevents a station very near the solution from dominating the sum and should be set to about half the typical spacing of stations so that the weighting of the nearest stations to the solution are similar throughout the network. The term *F* ensures that there is a minimum, *M*_*min*_, of fitting arrival times and is defined

$$\begin{array}{l} F(x,t^{\prime })=1\;\mathrm{if}\;\overset{N}{\underset{i=1}{\sum }}f_{i}(x,t^{\prime })\geq M_{\min}\\ F(x,t^{\prime })=0\;\mathrm{otherwise} \end{array}$$ (8)

The two approaches of maximizing Eqs (3) and (6), which we term methods 1 and 2, respectively, both provide a set of fitting detections. Eq (1) is then utilized to construct a likelihood function based just on these detection times, and this is integrated over the origin times that are consistent with the master detection time to construct a spatial probability distribution function

$$P(x)=K\int_{-m\sigma_{1,1}}^{{m\sigma }_{1,1}}\prod_{i=1}^{M}exp[-\frac{1}{2\sigma_{i}^{2}}{\left(T_{i}-{\hat{t}}_{i}(x)-O(x)-t\prime \right)}^{2}]dt\prime$$ (9)

where *K* is a normalization term chosen so that the spatially integrated probability density is unity and *M* the number of fitting detections.

For simplicity, solutions are obtained based on a single travel time versus distance curve. The ray tracing program BELLHOP [30] is used to calculate ray paths assuming a water depth of 2600 m, a source at 25 m depth [31], and a water column velocity model obtained from the NOAA World Ocean Atlas [32] for the month of December and the center of the network (46.5°N, 127.05°W). At each distance a single travel time is obtained by averaging the times observed for each path using their predicted amplitude as weights. A third order polynomial is fit to the travel times as a function of the straight-line source to station distance out to a distance of 300 km. Similar calculations with water depths ranging from 200 m to 3 km are consistent with this travel time curve to within ~1 s out to 200 km although the errors in the calculated times likely exceed this because our simplified approach does not account for the loss of energy due to attenuative interactions with the seafloor. To obtain solutions efficiently to Eqs (3) and (6), travel times from each station are precomputed onto a latitude-longitude grid that encloses the network and has a grid spacing of 0.005° in latitude (~550m) and 0.01° of longitude (~780 m). The integrals of Eqs (3) and (6) are obtained numerically with a temporal discretization of 0.25 s for master detection origin time misfit *t’*.

Table 2 lists the values of parameters used for the solutions. The localization methods require an estimate of the detection time uncertainty that must combine the uncertainty in identifying the time of its arrival and the uncertainty in modeling it. Because the first harmonic of the B call has only a gentle slope in frequency the detection time from cross correlation has a significant uncertainty which we estimate ranges from 1 s for high probability calls with probabilities of ≥0.9 to 2.5 s for calls with probabilities of ≤0.2. For the travel time predictions, we assume the uncertainty from fitting a smooth curve to the travel time predictions is 1 s. We estimate that the predictions themselves have an uncertainty that increases from ~1.25 s at 75 km (mostly higher probability detections) to 2.5 s at 150 km (lower probability detections) based on the standard deviation of the weighted distribution of travel times calculated by BELLHOP that contribute to the predicted time. The resulting total detection time uncertainty is thus estimated to vary from 2 s for detections with probabilities of ≥0.9 to 3.5 s for detections with probabilities of ≤0.2 (Table 2).

**Table 2:** Localization parameters.

Parameter	Value
Minimum probability for a master detection	0.9 (also consider 0.8 and 0.95)
Minimum probability for a detection in the solution	0.2 (also consider 0.1 and 0.5)
Maximum distance of a station from the station of the master detection	150 km
Maximum time of a detection after the master detection	100 s
Maximum allowed master detection misfit normalized to its uncertainty, *m*	3
Detection time uncertainty, *σ*,(based on probability, *p*)	2 s (*p* ≥ 0.9)
	2.5 s (*p* = 0.8)
	3 s (*p* = 0.5)
	3.5 s (*p* ≤ 0.2)
Minimum contribution to the likelihood product of Eq (3) for a poorly fitting station, *q*_*small*_	exp(-4.5) = 0.011
Maximum allowed detection time misfit normalized to uncertainty of a fitting arrival time in Eq (5), *m’*	3
Minimum distance from the station to the call in the calculation of Eq (6), *R*	50 km
Minimum number of stations for a solution, *M*_*min*_	4

For the two methods, we apply Eqs (3) or (6) to find a preferred set of detections. If there are at least 4 detections fitting to within three times their estimated uncertainty, we compute the probability density function *P*(***x***) in Eq (8). If the 95% confidence limits obtained from selecting the appropriate contour level in *P*(***x***) define two or more distinct maxima or extend to the side of the localization grid, or if the 1-σ horizontal uncertainty exceeds 20 km in any direction, the solution is discarded. For acceptable solutions, we use spline interpolation to find the location, x* that maximizes the probability density to a precision of 100 m. We also compute the components of mean location $\bar{x}$ and covariance matrix **C** from of *P*(***x***) according to

$${\bar{x}}_{i}=\iint P(x_{1},x_{2})\;x_{i}\;dx_{1}\;dx_{2}$$ (10)

and

$$C_{ij}=\iint P(x_{1},x_{2})(x_{i}-{\bar{x}}_{i})(x_{j}-{\bar{x}}_{j})\;dx_{1}\;dx_{2}$$ (11)

where *x*_1_ and *x*_2_ are the two horizontal coordinates that form ***x***, and *i* and *j* have values of 1 or 2. In general, the mean location and most probable location are very similar with the difference much smaller that the uncertainty obtained from the covariance matrix.

### Fitting smooth tracks

When calls are grouped into sequences from the same whale, it can be useful to fit a smooth track that accounts for the uncertainties in locations to estimate the net speed and direction of motion. For three calls on a track, a smoothing constraint that penalizes changes in speed and direction can be written

$$(1-w)(x_{i-1}-\delta x_{i-1})-(x_{i}-\delta x_{i})+w(x_{i+1}-\delta x_{i+1})=0$$ (12)

Where **x**_i_ is the current horizontal location of the *i*^th^ call, δ**x**_i_ is the change in location from smoothing, and the weighting is based on the origin times, *O*, of the calls according to

$$w=\frac{O_{i}-O_{i-1}}{O_{i+1}-O_{i-1}}$$ (13)

In order to provide uniform smoothing along the track, two weights must be applied to Eq (11). The first, 1/(*O*_*i*+1_−*O*_*i*−1_), strengthens the penalty when the calls are closer in time and thus, uniformly penalizes change in the rate of change of speed and direction. The second, 1/min(*w*, 1−*w*), accounts for the fact that when the central location is close in time to one of the outer points, a given change in the position of the central call will result in larger changes in speed and/or direction.

The weighted constraints of Eq (11) can be written for all calls in the track

SδX=−SX (14)

where **X** and δ**X** are vectors that include the current locations and changes in location of all the calls in the track, respectively, and matrix **S** applies the smoothing weights. The normalized spatial misfit of the *i*^th^ smoothed location relative to the solved location $x_{i}^{*}$ is

$$C_{i}^{-\frac{1}{2}}(x_{i}+\delta x_{i}-x_{i}^{*})$$ (15)

where **C**_*i*_ is the spatial covariance matrix of the solved location.

A solution that seeks to fit the locations of all calls with a smooth track can then be written as

$$\left[\begin{array}{c} C^{-\frac{1}{2}}\\ \alpha S \end{array}\right]\delta X=\left[\begin{array}{c} C^{-\frac{1}{2}}(X^{*}-X)\\ -\alpha S\;X \end{array}\right]$$ (16)

where **C** is the covariance matrix of all locations and α is the weighting of the smoothing constraint. This is an overdetermined weakly non-linear inverse problem that can be solved iteratively using a least squares method [33] with **X** updated by adding δ**X** after each iteration until the solution converges. If the spatial covariances of the locations are properly scaled, then the smoothing weight can be steadily decreased until the locations are fit to their expected uncertainty. If necessary, locations that are obvious outliers because they cannot be fit by the smooth track, can be removed and the inversion repeated.

An alternative approach to fitting a smooth track is to combine the smoothing constraint of Eqs (11)–(13) with the double difference method [34]. This approach was developed for earthquake locations, but it is well suited for refining tracks of marine mammals with stereotyped calls and its application to fin whales has been described in detail by Wilcock [13].

Rather than seeking to minimize travel time residuals, the double difference method seeks to adjust locations so as to fit the difference between the residuals on stations that are common to two nearby calls. A double difference time, *d*, can be written

$$d_{i,jl}=(T_{i,j}-T_{i,j}^{pred})-(T_{i,l}-T_{i,l}^{pred})$$ (17)

where *i* is the station index, and *j* and *l* are the indices of nearby calls. This double difference time can be equated to changes in the call locations and origin times according to

$$d_{i,jl}=\frac{\partial {\hat{t}}_{i,j}}{\partial x}\delta x_{1,j}+\frac{\partial {\hat{t}}_{i,j}}{\partial y}\delta x_{2,j}+\delta O_{j}-\frac{\partial {\hat{t}}_{i,l}}{\partial x}\delta x_{1,l}-\frac{\partial {\hat{t}}_{i,j}}{\partial y}\delta x_{2,l}-\delta O_{l}$$ (18)

where $\hat{t}$ are the predicted travel times and δ*x*_1_, δ*x*_2_ and δ*O* changes in the horizontal coordinates and the call origin time. A set of double difference times can be written in matrix form as

$$G\left[\begin{array}{c} \delta X\\ \delta O \end{array}\right]=d$$ (19)

where **G** is a sparse matrix of partial derivatives, δ**O** a vector of changes in origin times, and ***d*** a vector of double difference times. If the uncertainties of the double difference times are known each row of **G** and **d** can be weighted by the reciprocal of the uncertainty. A solution that combines the double difference times with the smoothing constraints of Eq (13) is then written as

$$\left[\begin{array}{c} G\\ \beta S\;0 \end{array}\right]\left[\begin{array}{c} \delta x\\ \delta O \end{array}\right]=\left[\begin{array}{c} d\\ -\beta S\;x \end{array}\right]$$ (20)

where *β* is the smoothing weight. As for Eq (15), this can be solved iteratively by least squares. After each iteration, the locations and origin times are updated, and the difference times are recalculated. Those difference times with absolute values that exceed a threshold of a multiple of the difference time uncertainty can removed from the next iteration. The advantage of the double difference method is that it is largely insensitive to errors in modeling the travel times because those errors are mostly common to the two calls being considered. It also provides a means to eliminate spurious detections because these can be expected to yield spurious difference times when paired with a correct detection or another spurious detection. The disadvantage of the method is that the smoothing weight cannot be adjusted so as to yield smoothed locations that match the location error, although it can be adjusted so that the difference time residuals match their uncertainty.

## Results and discussion

### Localizations

**Fig 5:**
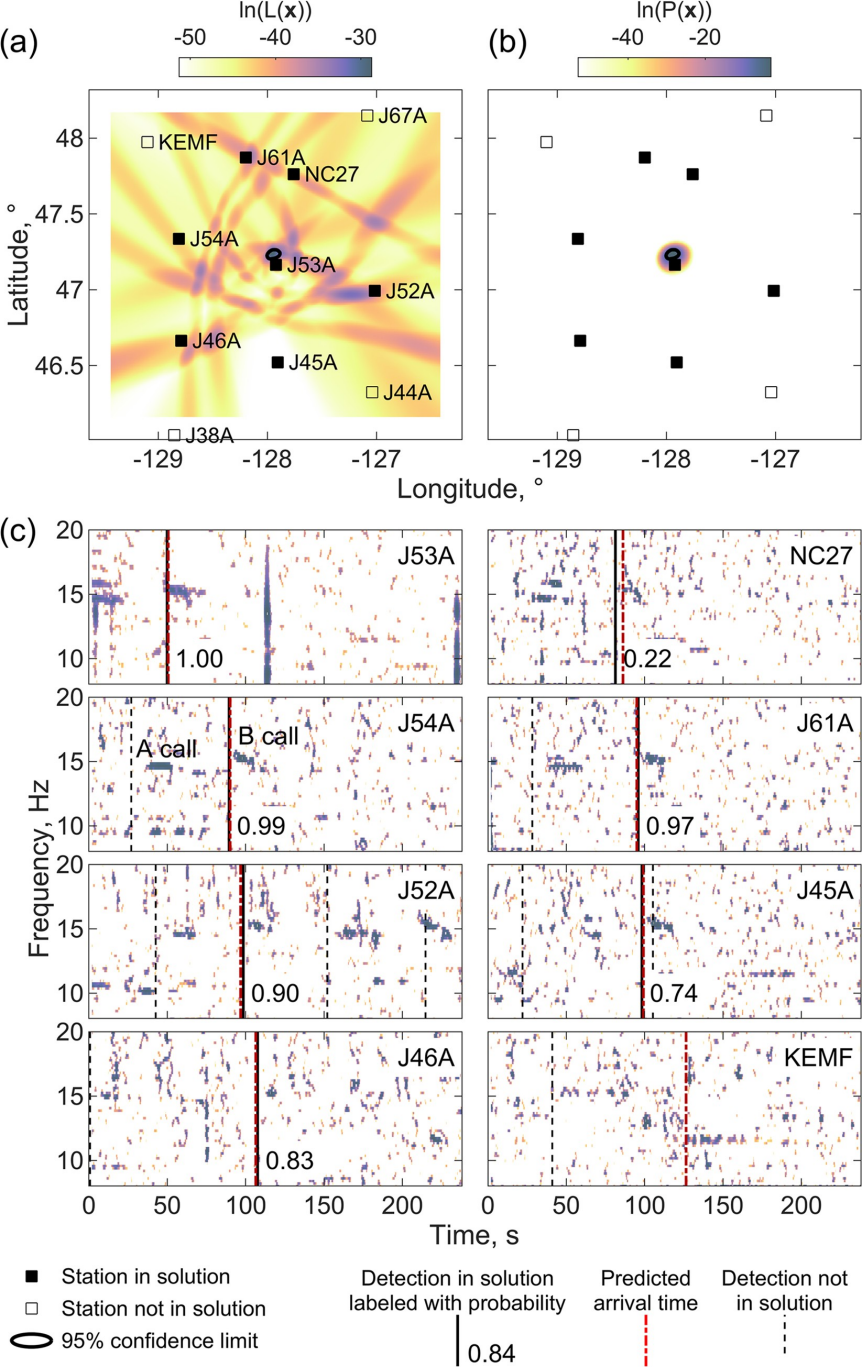
Correct localization with method 1. An example of a successful localization using method 1 that used the likelihood function of Eq (3) for a master detection on station J53A at 00:05:35 UT on December 13, 2011. The upper two panels are maps showing (a) the logarithm of the likelihood function **L**(***x***) (Eq 3) and (b) the probability density function *P*(***x***) (Eq 8) with a color scale. Labeled squares show stations, with filled squares indicating stations with detection times in the solution. The 95% probability area of the location is shown by a bold black contour. (c) Eight spectrograms for 4 minutes of data starting one minute before the master detection with detections used in the solution shown as solid black labeled with the estimated probability the detection is a true positive, other detection times as thin dashed black lines, and the predicted arrival times as dot-dashed red lines. Spectrograms are created with a 4-s-window and 95% overlap, normalized by subtracting the median at each frequency, and shown as a color scale for values that exceed the 90^th^ percentile value. Example A and B calls are labeled on the spectrogram for station J54A.

To evaluate the localization algorithm, we manually inspected spectrograms and the timing and peak recognition scores of detections for example solutions to determine whether the detections in the location were unambiguously from a single call. For a sequence of calls, this involved determining whether the detections were from the same call within the sequence and assessing whether stronger detections not included in the solution were from different calls within the sequence or could be unambiguously assigned to a call from a whale at a different location. For calls not within sequences, the manual assessment was harder and involved determining whether subsets of detections within the solution could be combined with other detections to yield another feasible location with higher probability detections at the earlier recording (nearer) stations. Based on this analysis we categorized localizations as correct, incorrect or uncertain. Fig 5 shows an example of a correct localization based on method 1 of Eq (3) for a master detection on station J53A. Although the likelihood function is quite complex because of the presence of 17 detections on 12 stations, the maximum value of the likelihood function leads to a location near station J53A that fits detections on 6 surrounding stations, 5 of which are high probability. The location has a formal 1-σ uncertainty of 1.9 km in longitude and 1.3 km in latitude.

**Fig 6:**
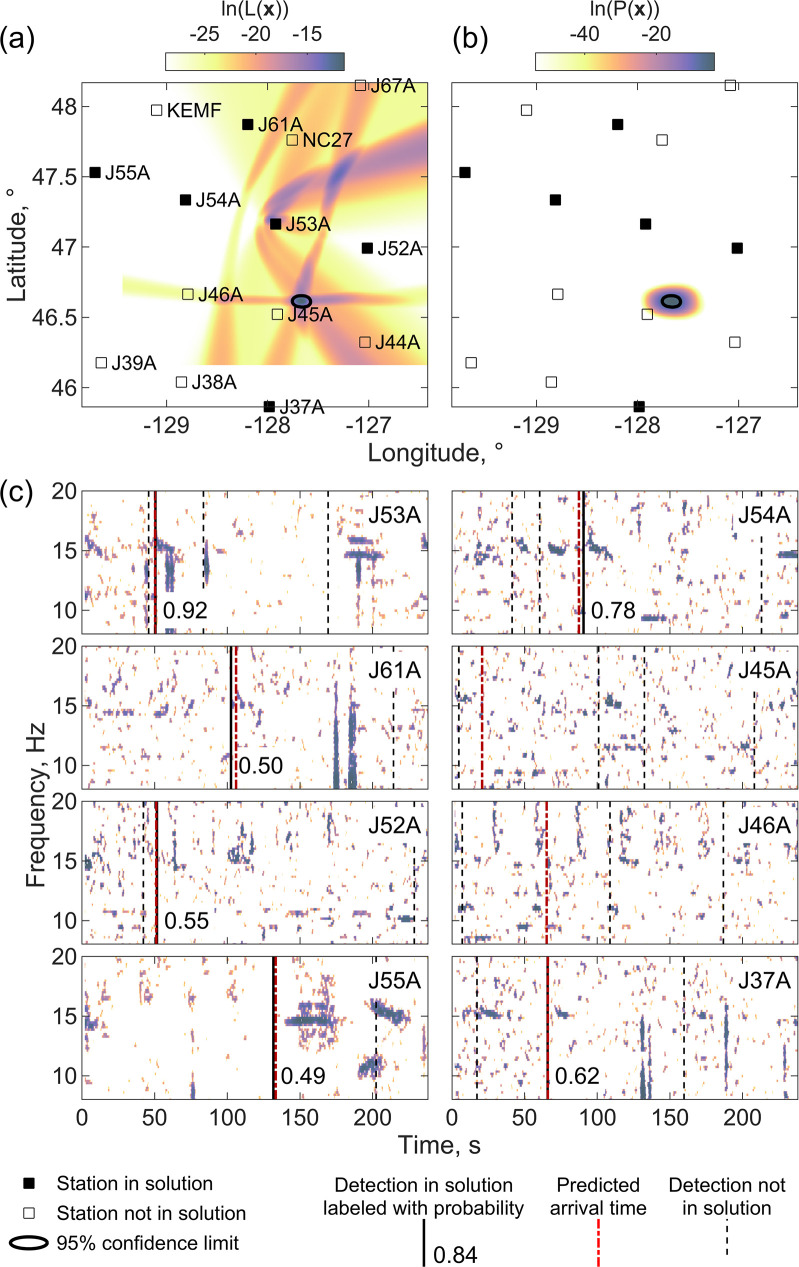
Incorrect localization with method 1. An example of an incorrect localization obtained with a master detection on station J53A at 00:02:30 UT on December 13, 2011, obtained using method 1 plotted using the same conventions as Fig 5.

In contrast, Fig 6 shows a localization for a call in the same calling sequence where method 1 fails. The chosen location models the correct detections for stations J53A (the master detection), J54A and J61A but incorporates erroneous detections for stations J52A, J55A and J37A and does not model detections on stations J45A and J46A. The chosen location lies well to the south of the correct location near J53A and is very close to station J45A which has no modeled detection. The formal location uncertainties are 2.9 km in longitude and 1.9 km in latitude. The correct solution near station J53A does appear as a local maximum in the likelihood function that fits the same number of detections to three standard deviations but is not selected because the fit is worse.

**Fig 7:**
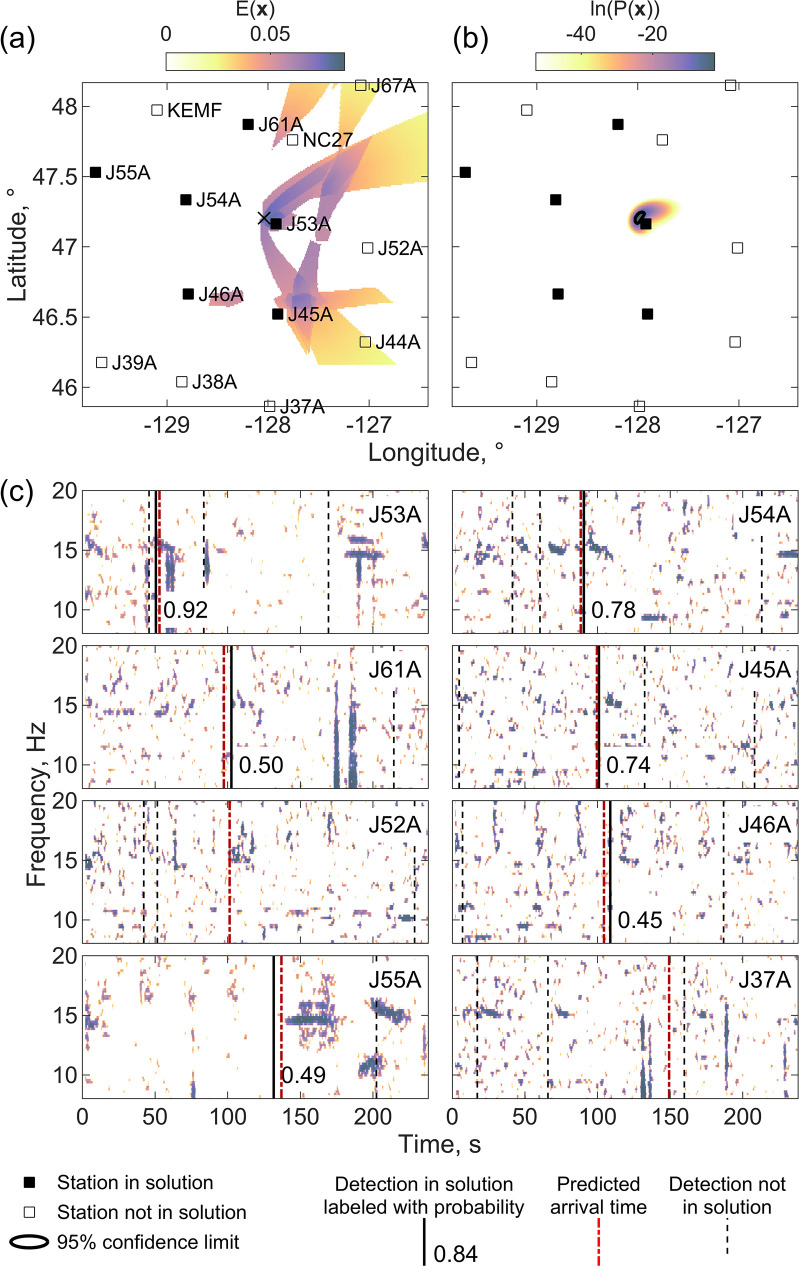
Correct localization with method 2. A correct localization for the same master detection as Fig 6 obtained using method 2 based on Eq (6) that maximizes the number of high probability nearby detections. The upper panels show (a) the function *E*(***x***) of Eq (6) with the highest value shown by a black x-mark, and (b) the probability function of Eq (8) plotted using the same conventions as Fig 5. The lower panels show spectrograms plotted using the same convention as Fig 5.

Applying method 2 of Eq (6) (Fig 7) leads to the correct solution because detections that are fit to within 3 standard deviations are weighted based on their probability and proximity. The final solution correctly predicts detections at 6 of the 7 closest stations and has a formal uncertainty of 1.8 km in longitude and 1.4 km in latitude. Interestingly, both solutions incorporate a spurious detection at station J55A which appears to be linked to a high amplitude A call. For the solution shown in Fig 7, this erroneous detection time is not particularly well fit. Because the predicted detection time is too late, it acts to pull the solution to the west.

**Fig 8:**
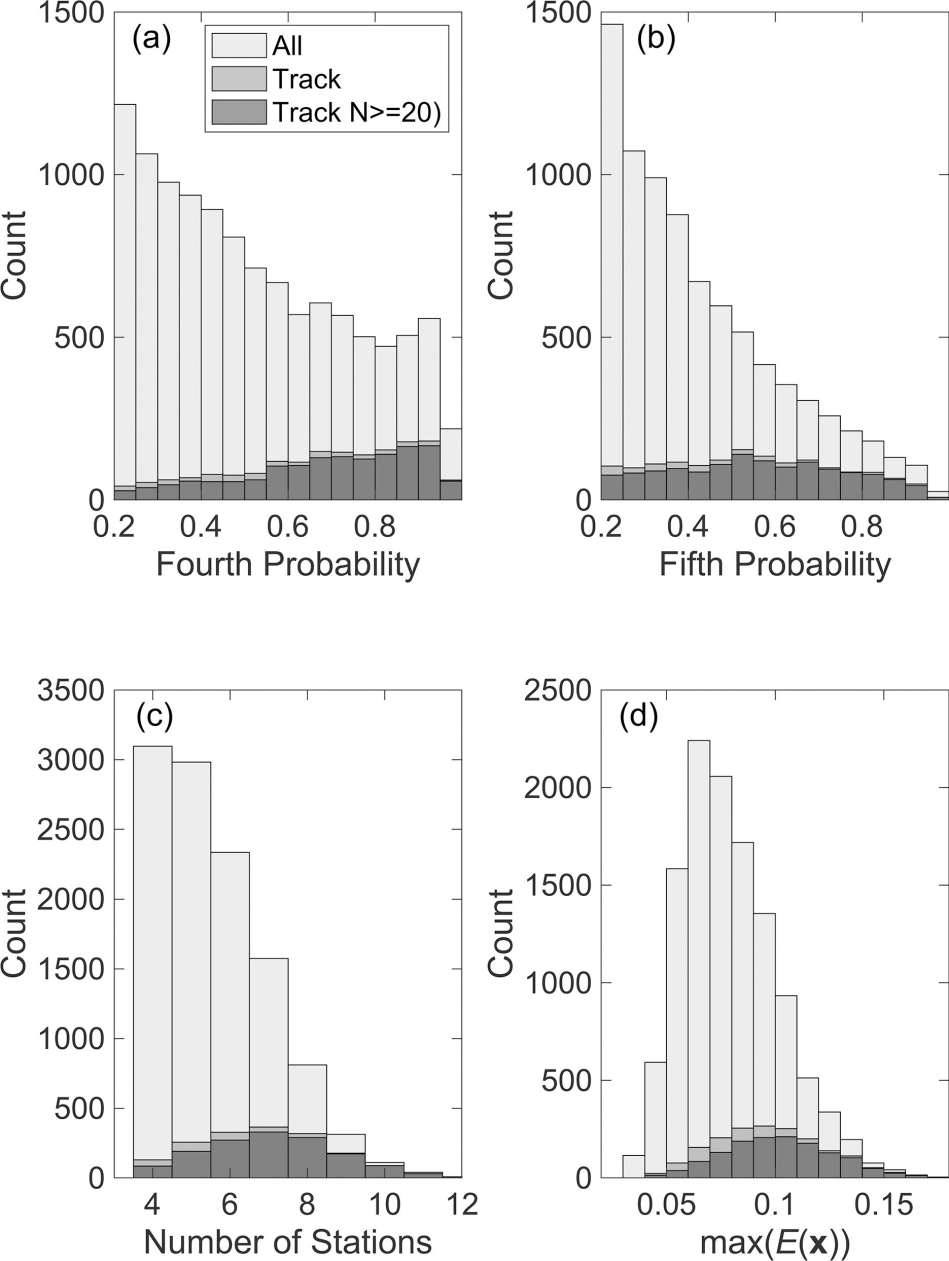
Characteristics of localizations. Histograms showing characteristics of the solutions for a 3-day interval from December 13 through December 15, 2011, obtained with a minimum master detection probability of 0.9 and a minimum detection probability of 0.2. (a) The estimated probability of the 4^th^ most probable detection in the localizations from method 1 (Eq 3) shown for all locations, locations within tracks and locations in tracks with at least 20 calls. (b) As for (a) but for the 5^th^ most probable detection for the subset of solutions with at least 5 detections. (c) The number of stations in the solution obtained with the method 1. (d) The maximum value of *E*(***x***,*t*′) (Eq 6) for localizations from method 2 which favors detections with high probabilities near the location.

Provided there are enough detections, both localization methods will yield bad or biased locations if they include spurious detections that are either false positives or a result of mixing true positives from more than one B call. The problem of false positives is well illustrated by examining some of the statistical properties of the locations for the 3-day test period. In Fig 8A and 8B, we show histograms of the probability of the fourth and fifth most likely detections in the localizations. At least 4 stations are required to locate a call in two horizontal direction and time [35]. However, the estimated probability of the fourth most likely detection (Fig 8A) is less than 0.5 for 52% of the solutions and less than 0.3 for 20%. A fifth detection adds redundancy to a solution that in the absence of many spurious detections can be used to identify an outlier, but 69% and 31% of such detections have probabilities less than 0.5 and 0.3, respectively (Fig 8B). Only 45% of the locations are based on more than 5 detections (Fig 8C), but as Fig 6 illustrates, the presence of 6 modeled detections is insufficient to guarantee a correct solution. If it is assumed that a reliable localization requires at least 6 modeled detections of which most are high probability, then only 12% and 5% of the solutions meet this requirement when the a fourth highest probability is required to be >0.8 and >0.9, respectively. If the fifth highest probability is required to meet these thresholds, the percentages are reduced to 4% and 1%, respectively. For the second localization method a value of *E* > 0.1 can be achieved with 6 high probability detections within ~70 km. Only 12% of the locations obtained with the second method meet this criterion (Fig 8D) and when the probabilities of the 4^th^ and 5^th^ detections are also required to exceed 0.8, this percentage is reduced to 8% and 3%, respectively. Even with very stringent criteria for accepting individual localizations, the false locations that arise from mixing true positives from different calls are not eliminated. This data set may be particularly prone to the problem of mixing two calls from the same whale because the 70 km spacing of OBSs is almost equivalent in travel time to the observed spacing of ~50 s of successive B calls in ABB-type songs [36].

**Fig 9:**
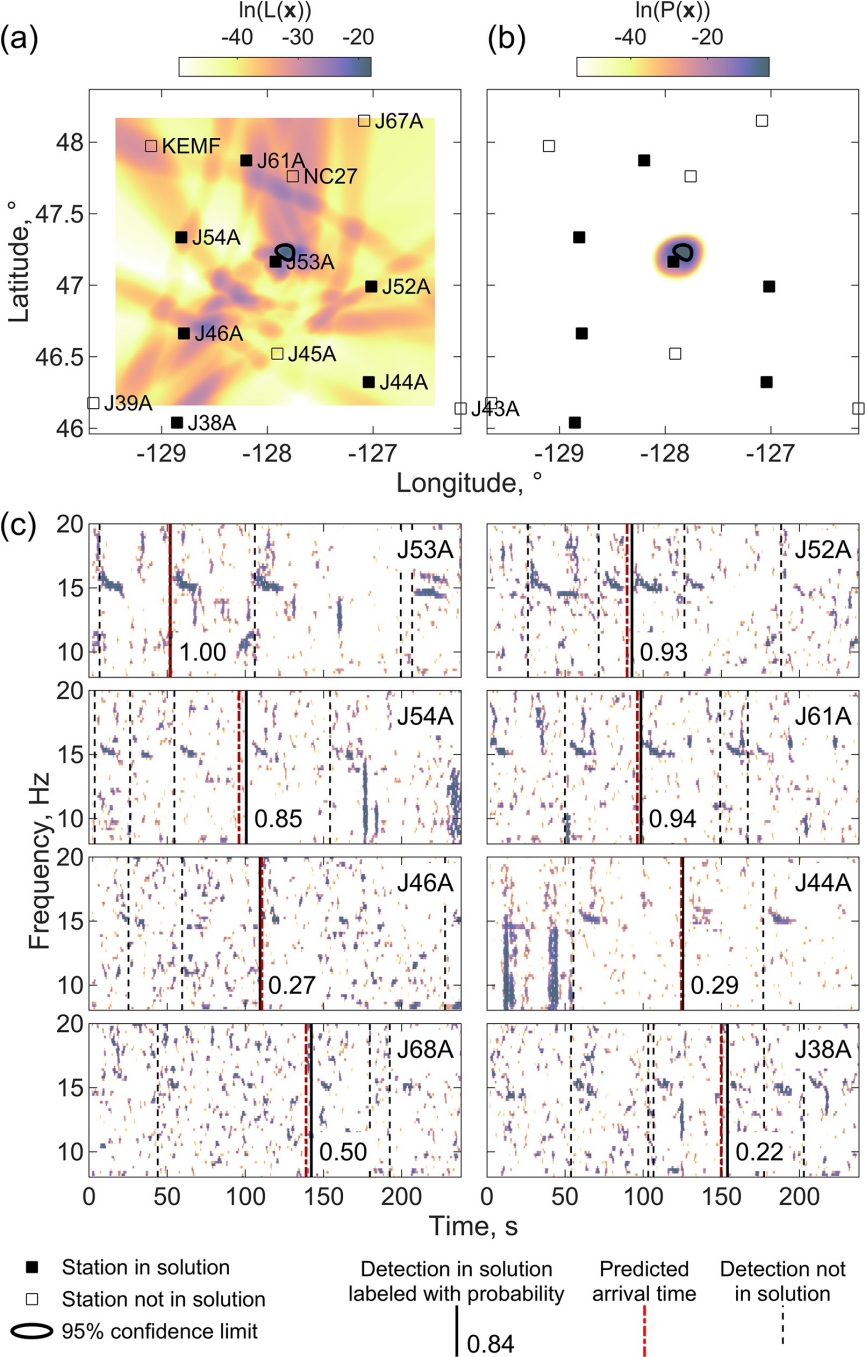
First ambiguous localization. A localization plotted using the same conventions as Fig 5 obtained using Eq (3) with a master detection on station J53A at 04:24:41 UT on December 15, 2011, that fits 9 detection times.

**Fig 10:**
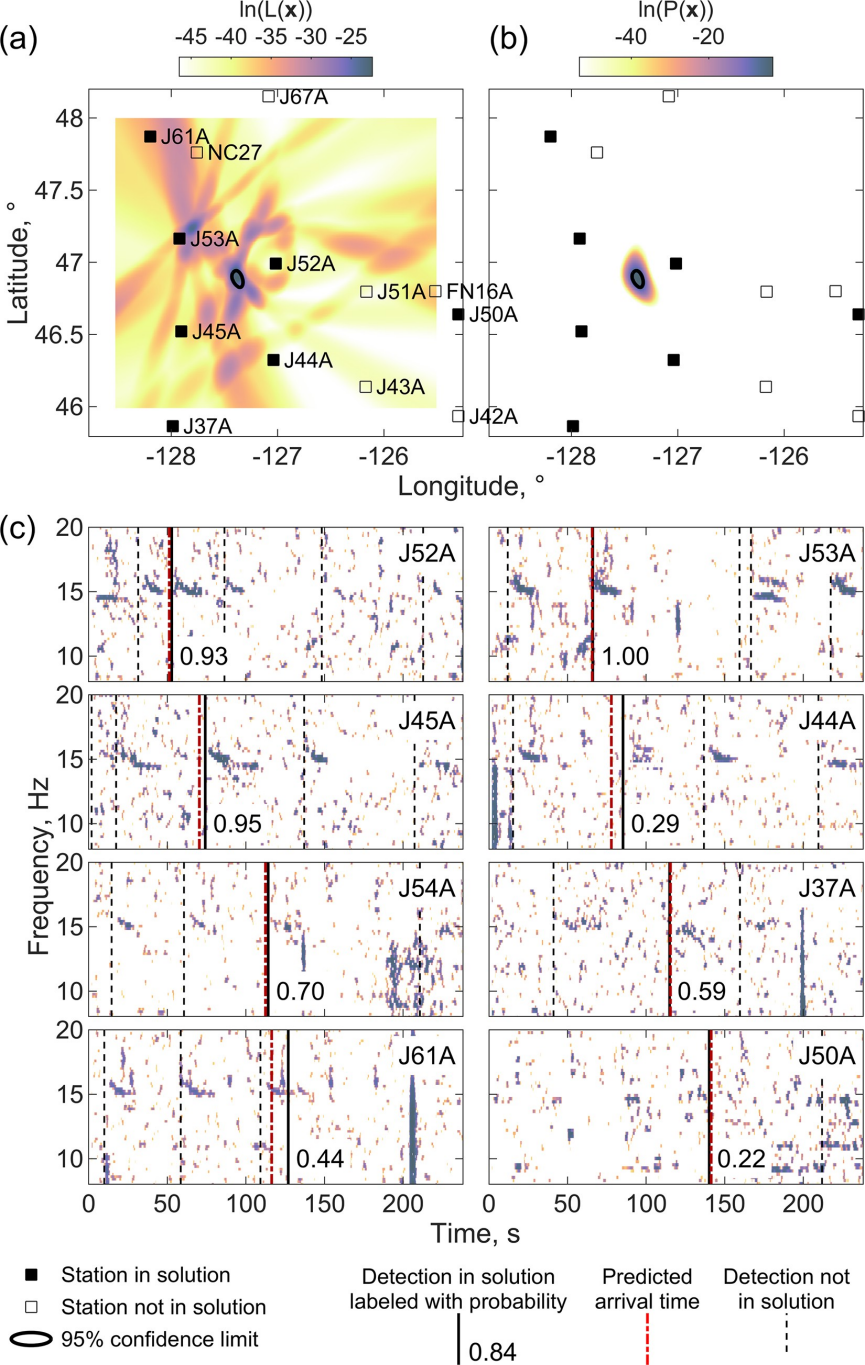
Second ambiguous localization. A localization plotted using the same conventions as Fig 5 obtained using Eq (3) with a master detection on station J52A at 04:25:22 UT on December 15, 2011, that fits 8 detection times. This solution incorporates the same detections for station J52A and J44A as Fig 9, but otherwise fits a different set of detections.

Figs 9 and 10 show an example where two alternative localizations are obtained using the same high probability (0.93) detection on station J52A but with different high-probability (1.00) detections on station J53A which appear to be successive calls from the same whale. Both solutions also fit a low probability detection (0.29) on station J44A but otherwise fit a completely different set of detections, many of which appear to be blue whales. From inspection of the spacing of calls in the spectrograms, it is clear that there are multiple whales calling during this interval which makes it difficult to tell which, if either, of the two locations is correct.

### Tracks

Because it is infeasible to inspect all solutions and sometimes difficult even after inspection to fully ascertain whether a given location is correct, the localization results are most useful for finding groups of locations that are consistent with a track for a single whale calling repeatedly. To do this, it is first necessary to discard repeat locations, which are defined as localizations that include a set of detections that are a subset of those for a localization obtained with an earlier master detection. A simple space and time filter is then applied to link locations into groups by searching for a location that is within 10 km and 15 minutes of at least 4 other locations and then repeating this process iteratively for all the locations added to each group. To account for pauses in calling, groups are combined if they are separated by no more than 1 hour, and if at least 4 locations in one group are each within 20 km of 4 locations in the other group. These spatial requirements are quite relaxed to allow for localization errors.

The localization methods allow a detection to be included in multiple localizations and as illustrated in Figs 9 and 10, there is the potential for detections for calls in sequences with a consistent call spacings to be both located into the correct tracks and mislocated into spurious tracks by systematically combining detections from successive calls. An iterative approach is used to eliminate such spurious tracks based on the observation that the correct track will very likely include more calls. After completing the localization process and finding tracks, all the detections within tracks with a minimum number of locations are assigned to belong to the location in that track. When the detection is incorporated into locations within more than one track, it is assigned to the location in the track with most locations. The localization procedure is then repeated with the flagged calls excluded from all but their assigned location. For our test data set, we found that two iterations in which the minimum number of locations is first 25 and then 15, reliably eliminates spurious tracks.

**Fig 11:**
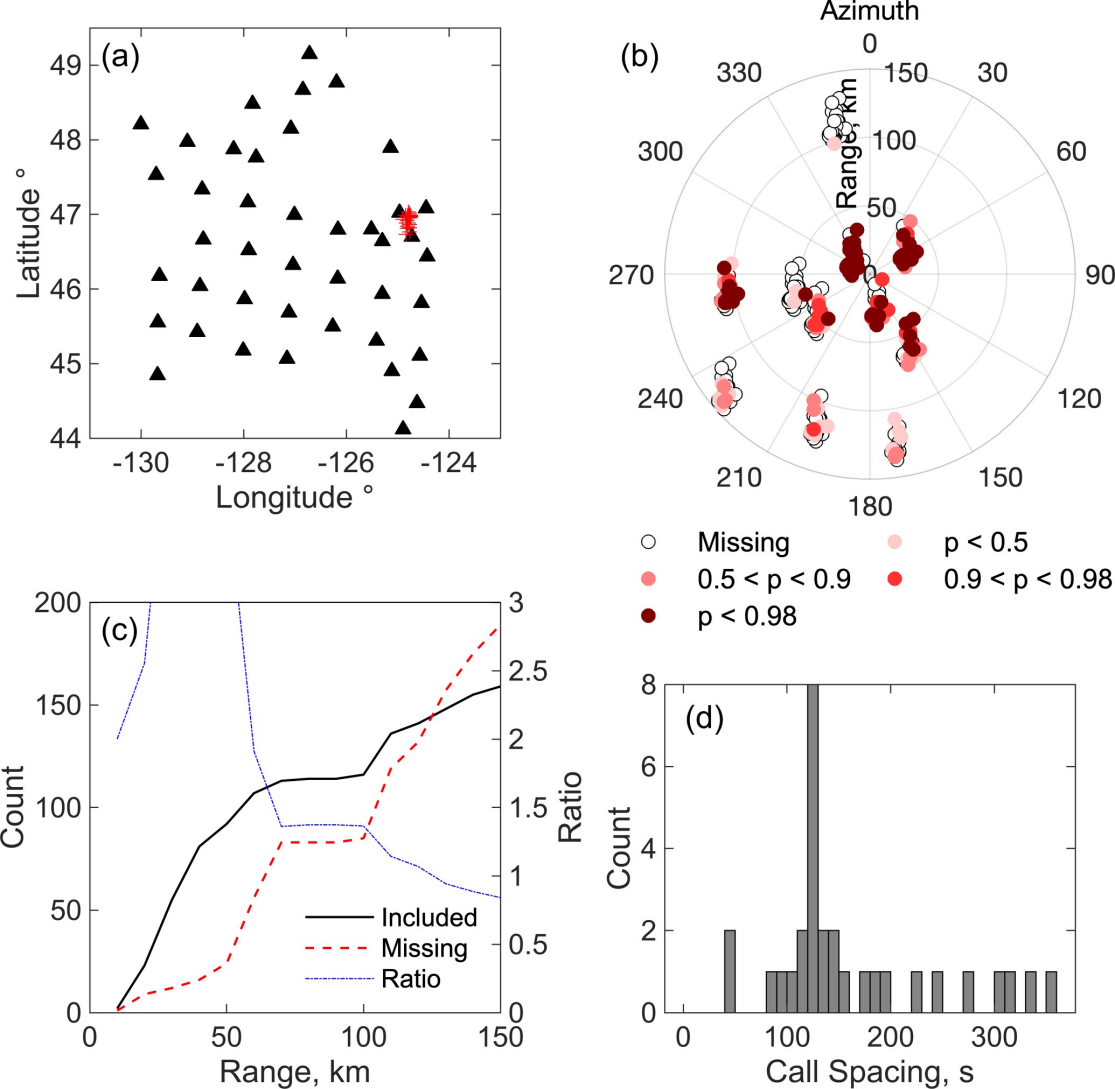
Characteristics of a reliable track. Some characteristics of an example track that is considered reliable, comprising 33 locations on December 14 between 1334 and 1519 UT. (a) Locations of calls in the track (red pluses) and seismic stations (black triangles). (b) Locations of stations that contribute detections to the localization of each call in the track plotted relative to the call location and color coded by the estimated probability that the detection is a true positive. Open circles indicate stations that are missing from the localization but that were recording detections as evidenced by the presence of at least one detection within a time window extending from 60 s before the first detection in the localization to 120 s afterwards. (c). Cumulative count of detections contributing to the call localizations as a function of the range to the location (black solid line) and of stations for which detections are missing in the localization but for which at least one detections is available at the station from 60 s before the first detection to 120 s afterwards (red dashed line). Also shown is the ratio of these two counts (dot-dashed blue line). (d) Histogram showing the time between of successive locations in the track.

**Fig 12:**
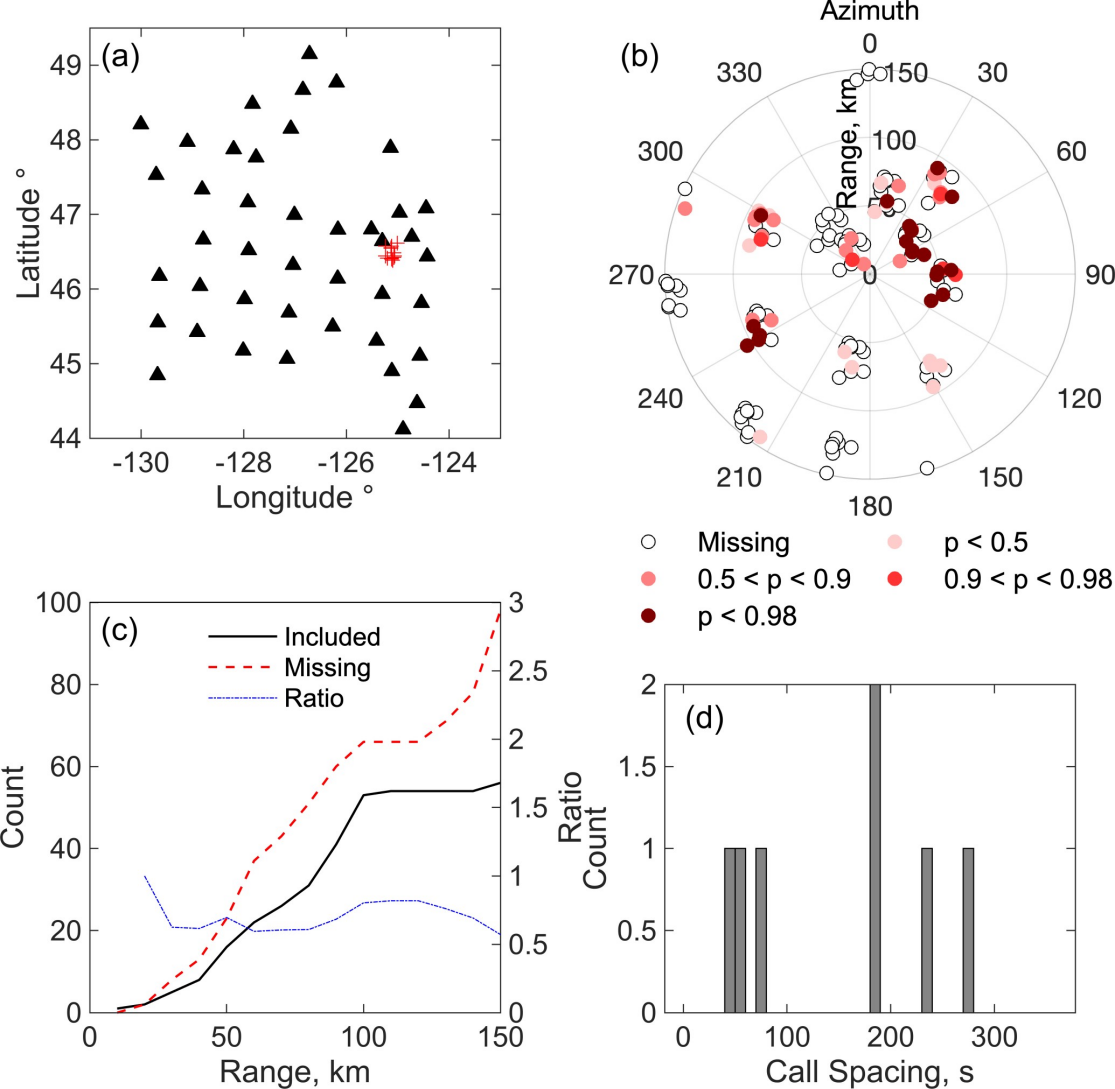
Characteristics of an unreliable track. As for (a) except for an example track that is considered unreliable, comprising 13 locations on December 13 between 0931 and 1117 UT.

Several approaches can be used to assess the quality of tracks, and these are illustrated by comparing two tracks on the eastern side of our network (Figs 11A and 12A), the first that we consider reliable (Fig 11) and the second that we do not (Fig 12). A reliable track should be characterized by higher probability detections at stations near the call locations, and stations near the call locations should generally have detections included in the locations. Figs 11B and 12B plot the locations of stations relative to each call location in the two tracks with color coding to illustrate stations with detection missing from the localizations and the probability of detections that are included in the localizations. In Fig 11B, the highest probability detections are mostly clustered around the locations and there are very few stations near the locations with detections missing from the localizations. In contrast, in Fig 12B, the higher probability detections are more scattered and there are many missing detections at nearby stations. Another way to assess the tracks is to plot the cumulative number of stations as a function of range (horizontal distance) from the call locations for both stations included in the localizations and stations that are missing. For reliable tracks, the ratio of included to missing detections is generally higher than one out to distances of at least 75–100 km (Fig 11C), while for spurious tracks it is generally less than one (Fig 12C).

The two primary Northeast Pacific Blue whale song sequences are AB songs in which A and B calls alternate with an inter-call interval between B calls of ~120 s and ABB songs in which each A call is followed by two or more B calls with an inter-call interval of ~50 s [36]. For a reliable track, a histogram of the time between successive locations will generally show a peak at one of these characteristic inter-call intervals (Fig 11D), while an unreliable track will not (Fig 12D). Finally, spurious tracks often yield infeasibly fast swimming speeds; the next section discusses how to estimate a smooth path and speed from the locations in a track. Based on applying these various approaches and looking at individual solutions, we infer that tracks with at least 20 locations are reliable in the test data set. Inspection of the characteristics of localizations (Fig 8) shows that the locations in tracks are on average significantly more robust than the full set of localizations.

Table 3 summarizes the results of fitting tracks to the 3-day test period with different choices of the minimum probabilities for the master detection and for including detections in the solution. For each choice of minimum probabilities, we evaluate four different processing schemes in which either the localization method is first used to assign detections to tracks and then either the same or the other method is used to obtain the final tracks. As the minimum probabilities are reduced, the number of locations within tracks increases as would be expected. Relative to a baseline solution with minimum master detection and detection probabilities of 0.9 and 0.2, respectively, decreasing the master detection probability to 0.8 or the detection probability to 0.1, leads to a ~20% increases in the number of locations in robust tracks with ≥20 locations while increasing the computation time (the number of detections that must be modeled) by ~40%. There is a clear benefit from assigning calls to the initial track locations with one localization method and then obtaining the final tracks with the other. This can be simply explained, because this approach will incorporate calls into a track if they are located successfully with either method. More locations are assigned to robust tracks when method 1 is used to initially assign detections to locations within tracks and method 2 then used to obtain the final locations.

**Table 3:** Results of processing data from December 13 through December 15, 2011.

Minimum Master Detection Probability	Minimum Detection Probability	Localization Attempts	Detections Modeled	Method to Assign Detections to Tracks	Method 1 used for Final Localizations		Method 2 used for Final Localizations	
					Calls in Tracks	Calls in Tracks (*N*_*T*_≥20)	Calls in Tracks	Calls in Tracks (*N*_*T*_≥20)
0.95	0.2	14,545	175,724	1	1077	876	1553	1245
				2	1368	1209	1398	1108
0.9	0.5	16,199	138,929	1	788	582	1180	922
				2	980	896	1080	810
	0.2	19,991	245,672	1	1274	1063	1981	1589
				2	1709	1473	1789	1378
	0.1	21,293	351,287	1	1607	1247	2504	1843
				2	2045	1612	2249	1680
0.8	0.2	26,778	335,419	1	1699	1305	2503	1949
				2	2056	1701	2234	1688

Method 1 and 2 refer to the localization methods that are based on using Eqs (3) and (6), respectively. *N*_*T*_ is the number of locations in tracks.

**Fig 13:**
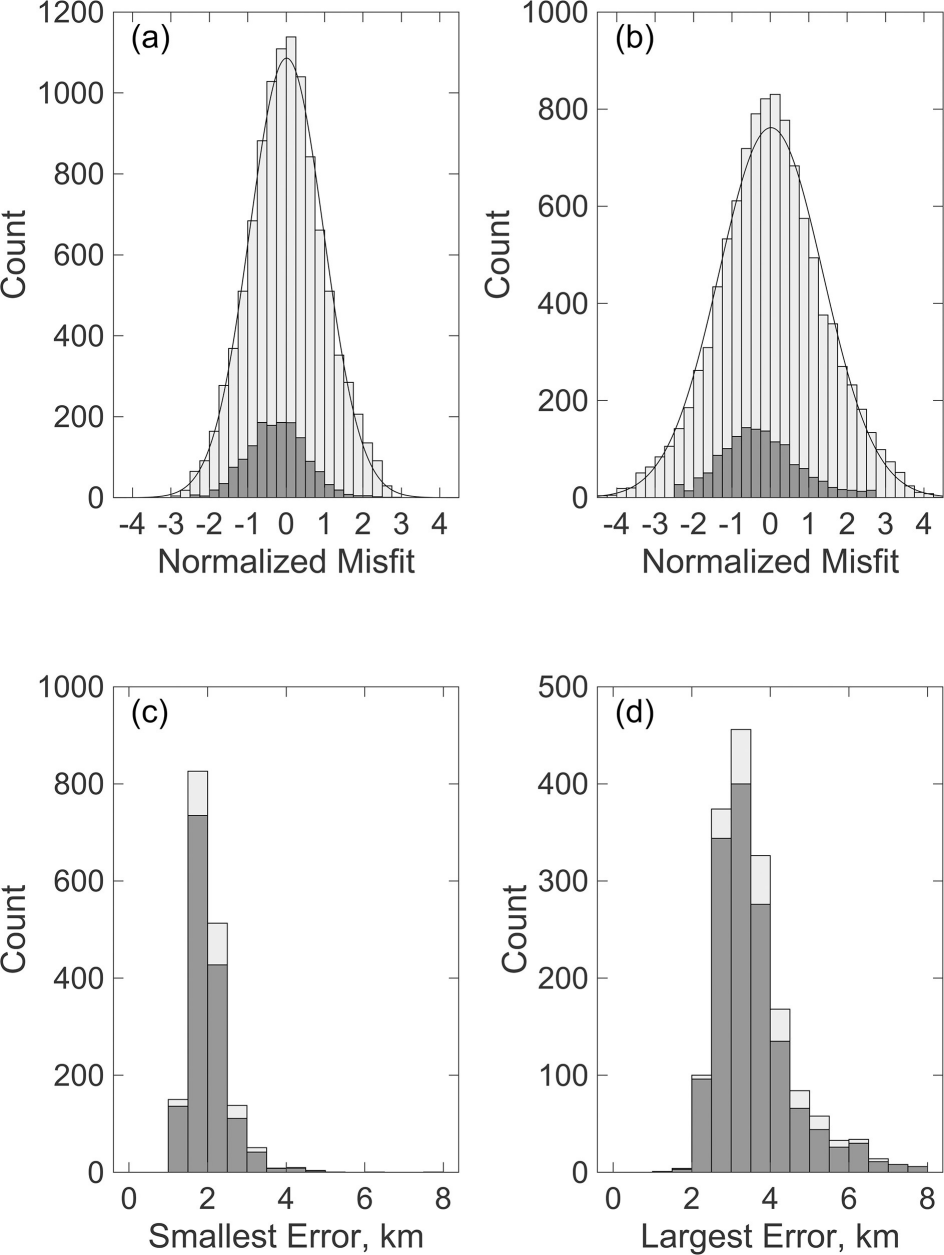
Statistics of locations in tracks. Characteristic of the locations within tracks obtained by first assigning detections to locations within tracks using one localization method and then using the other method to obtain the final locations. These plots are for a minimum probability of master detections and all detections of 0.9 and 0.2, respectively but the plots have similar characteristics for tracks obtained with all the choices of minimum probabilities listed in Table 3. (a) Histogram of the travel time misfits normalized to the assumed uncertainties (Table 1) for master detections (dark shading) and all detections (light shading) for locations in tracks with ≥ 20 locations with the final locations obtained using localization method 1 (Eq 3). A solid line shows a Gaussian distribution fit to the histogram which has a standard deviation of 0.96. (b) As for (a) except the final locations are obtained with localization method 2 (Eq 6). The Gaussian distribution fit to the histogram has a standard deviation of 1.35. (c) Histogram of the smallest horizontal 1-σ location error for all tracked locations (light shading) and tracks with ≥20 locations (dark shading) for final locations obtained with localization method 1. (d) As for (c) but for the largest horizontal location error.

Fig 13A and 13B shows the distribution of normalized travel times misfits for localizations in tracks based on final localizations obtained with method 1 and 2. Both distributions are fit well by a normal distribution, but the uncertainties for the case where the final track is obtained with localization method 2 are much larger. The problem with the localization method 2 is that while maximizing *E*(***x***) favors solutions with many probable and proximal detections, it also favors solutions which just fit true positives within the prescribed uncertainty threshold while also incorporating additional false positives. When the location is then obtained by maximizing Eq (8), the false positives broaden the distribution of misfits. For this reason, the preferred approach is to use localization method 2 to assign detections to the locations in tracks and then localization method 1 for the final locations. The typical location uncertainties are several kilometers (Fig 13C and 13D).

The variance of the travel time misfits normalized to their uncertainty, *s*^2^, can be written for tracked calls as

$$s^{2}=\sum_{j=1}^{Q}\sum_{i=1}^{M_{j}}\frac{\left(T_{i,j}-T_{i,j}^{pred}\right)}{\sigma_{i,j}^{2}}/\sum_{j=1}^{Q}M_{j}$$ (21)

where *j* is the index of the *Q* calls that are in tracks, *i* the index of *M*_*j*_ detections in each call location, *and T*^pred^ the predicted detection times for the most probable location given by

$$T_{i,j}^{pred}=O(x^{*})+{t^{\prime }}^{*}+{\hat{t}}_{i}(x^{*})$$ (22)

where t’^*^ is the origin time adjustment for the most probable location. If the assumed travel time uncertainties are correct, then the expected value of *s*^2^ is given by

$$E(s^{2})=\frac{\sum_{j=1}^{Q}M_{j}-p}{\sum_{j=1}^{Q}M_{j}}$$ (23)

where *p* = 3 is the number of free parameters (*x*_1_, *x*_2_ and time). For the example shown in Fig 13A, the expected value of *s* is 0.74 compared with the observed value of 0.96 which suggests that either that the uncertainties in Table 1 are underestimated or the solutions include spurious detections. We explored increasing the assigned detection uncertainties and this has little effect on the mismatch between the expected and observed values of the travel time misfits, suggesting that the cause is the presence of spurious detections in the solutions.

**Fig 14:**
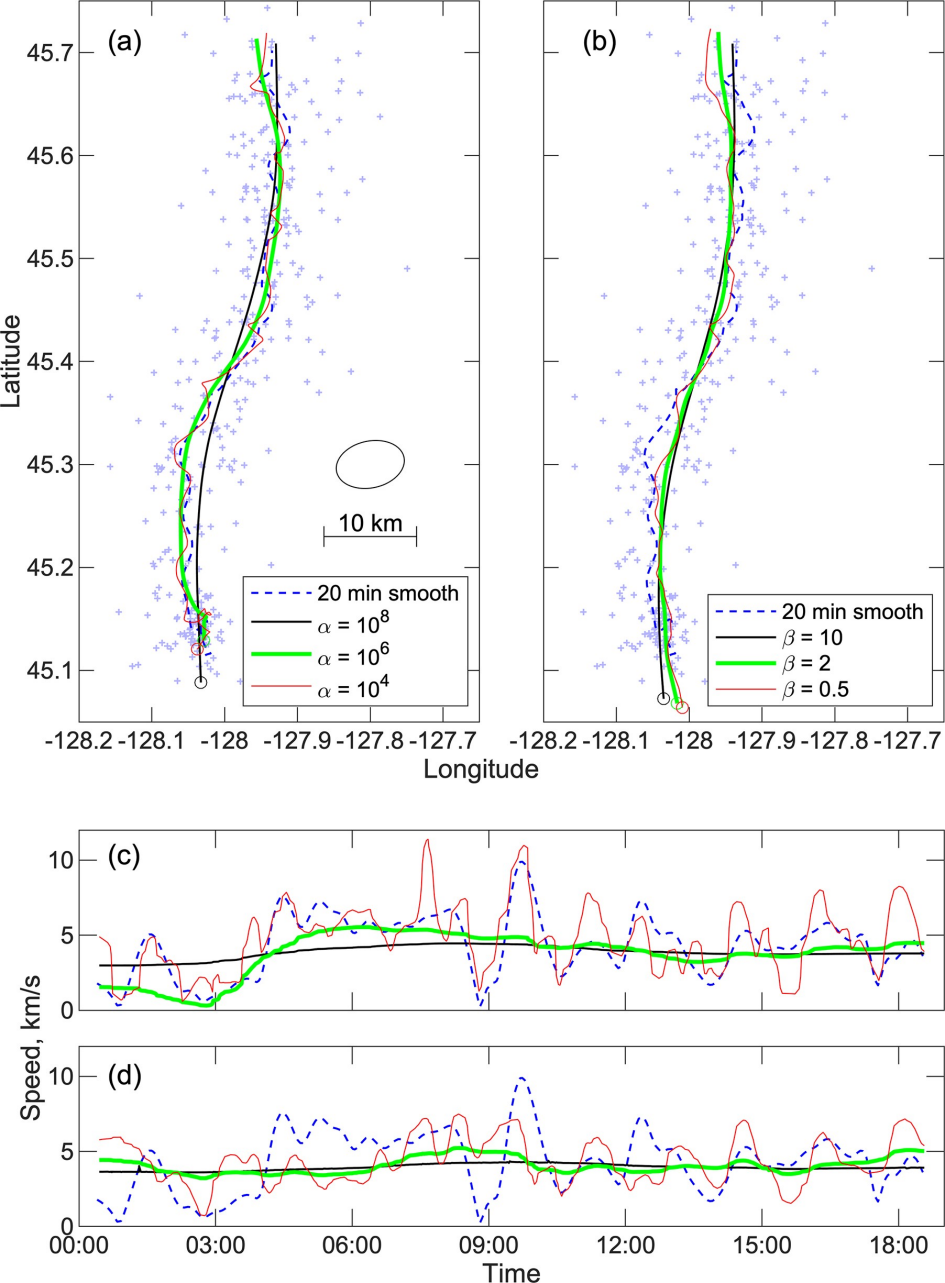
Smooth paths for a long track. An example of smoothed paths that are fitted to a track comprising 355 call locations between 0021 UT and 1834 UT on December 13, 2011. (a) Smoothed tracks obtained using the method of Eq (15) with three choices of smoothing weight after discarding the 21 outlying locations. Call locations are shown as faint plus symbols with an ellipse illustrating the average location uncertainty. The mean squared normalized spatial misfit is 2.49, 2.07 and 1.81 for smoothing weights α = 10^8^, 10^6^ and 10^4^, respectively. Also show is a smoothed track based on averaging locations temporally by weighting with a Gaussian function with a 20-minute standard deviation. (b) Tracks obtained using the double-difference method of Eq (19). The starting data set comprises 13,174 double-difference times and is constructed by linking each call to up to 8 calls within the next hour if there are four common stations. The solutions were obtained with 5 iterations at each smoothing weight and with poorly fitting double difference times discarded after 3 iterations if the absolute misfit exceeded three times the assumed uncertainty. Totals of 11,999, 11,683 and 11,625 difference times were used in the final solutions for smoothing weights of *β* = 10, 2, and 0.5, respectively. The mean variances of the double difference time misfits normalized to their assumed uncertainties are 0.58, 0.44, and 0.41. (c-d) Speed as a function of time corresponding to the tracks shown in (a) and (b), respectively.

**Fig 15:**
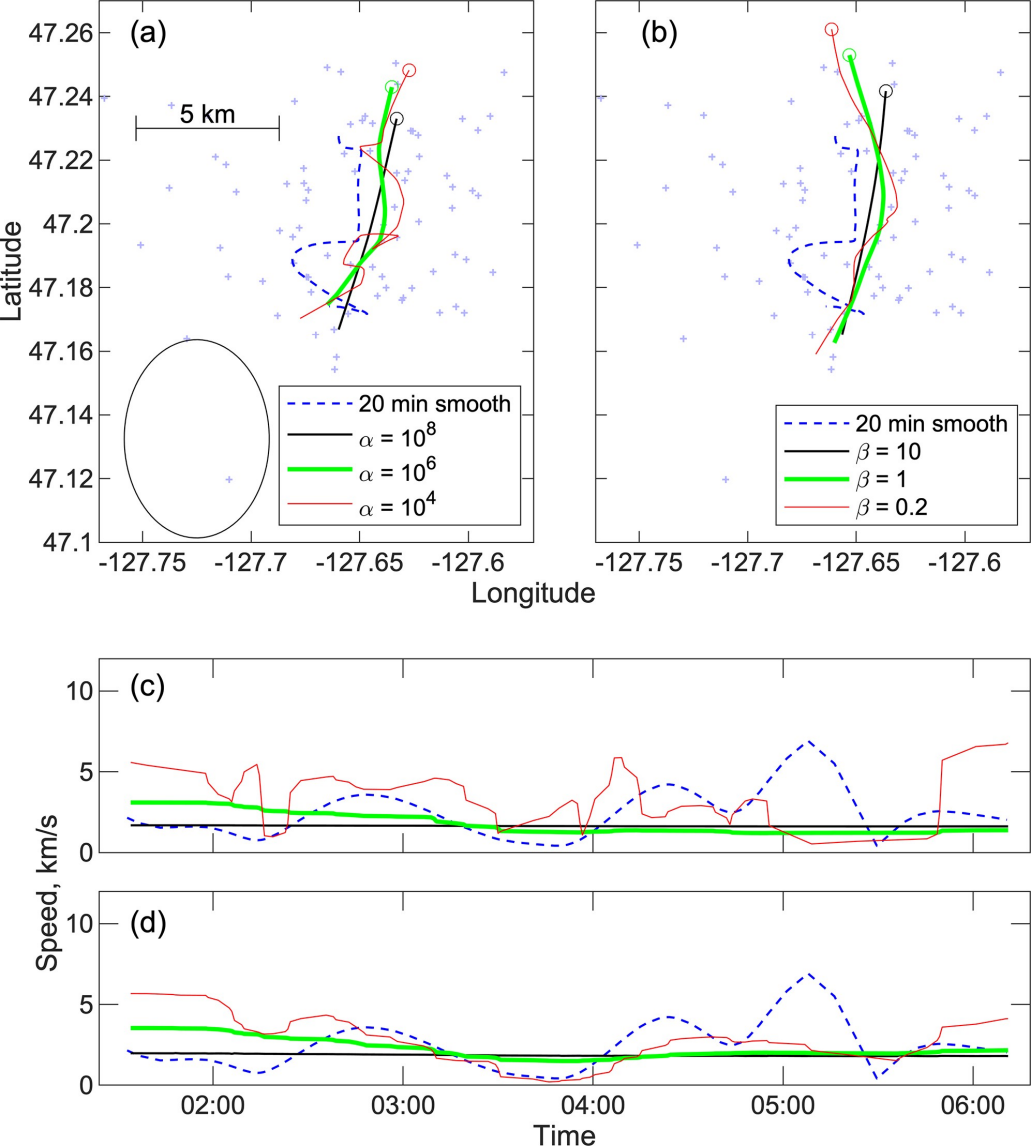
Smooth paths for a short track. As for Fig 14 except for a track with 92 locations from 0132 UT to 0611 UT on December 15, 2011. (a) Solutions are obtained after discarding 11 outlying locations and the mean squared normalized spatial misfit is 2.22, 2.14 and 1.97 for smoothing weights *α* = 10^8^, 10^6^ and 10^4^, respectively. (b) The starting data set comprises 3638 double difference times and 3204, 3161 and 3159 are used in the final solutions for smoothing weights of *β* = 10, 2, and 0.5, respectively. The mean variances of the double difference time misfits normalized to their assumed uncertainties are 0.44, 0.37 and 0.36.

The two methods of fitting a track are illustrated with two examples (Figs 14 and 15). The first track (Fig 14) is for a whale that swims about 70 km north over 18 hours at an average net speed of ~4 km/hr. The second (Fig 15) is a shorter track lasting under 4 hours in which the whale moves southward at ~2 km/hr. Both approaches are able to successfully fit the data with smoothed paths. For the first method of Eq (15), a chi-squared test is used to discard calls whose spatial location uncertainties are inconsistent with a smooth path at the 99% confidence level. The inference is that such locations are likely biased by spurious detections. After removing 21 poorly fitting locations out of 355, the track with a smoothing weight α = 10^6^ leads to a fairly smooth track that fits the locations with an average normalized spatial variance of 2.07, which is close to the expected value of 2 when the locations are fit to their uncertainty. For the 2^nd^ track, 11 locations out of 92 are discarded and the mean normalized spatial variance for α = 10^6^ is 2.14. The locations are fit to their uncertainty for α = 10^4^, but the track is quite complex suggesting that the location uncertainties are underestimated because of the presence of spurious detections or that the whale track is indeed quite complex.

For the double difference method of Eq (19), the solutions for each track with three choices of smoothing weight are based on between 87% and 91% of the double difference times which suggests that about 5% of the detection times are bad presumably because they are for detections that are not from the call. For these solutions, we assume that the variance of the difference times is the sum of the variance for the two observed detection times. This can be expected to be an overestimate since the double difference times will eliminate most of the travel time modeling uncertainty because the ray paths are similar. For the middle smoothing weight, the mean variances of the double difference time misfits normalized to their assumed uncertainties are 0.44 and 0.36 for the two tracks, respectively. This suggests that over half the uncertainty in the detection times is related to errors modeling travel times which is consistent with our assumptions in assigning travel time uncertainties.

## Conclusions

We have described an automated algorithm to locate the B calls of the northeast Pacific blue whale using time of arrival that has been developed to analyze data from a network of widely spaced ocean bottom seismometers deployed for the Cascadia Initiative experiment off the coast of the Pacific Northwest. Detections are based on spectrogram cross-correlation using a template that matches the first harmonic of the B call. A probabilistic approach is used to find locations that are consistent with the times of a sufficient number of detections following a strong master detection. Because the receivers are spaced at up to 70 km apart, the algorithm must consider feint detection that have a significant probability of being false positives. False localizations frequently result from including false positives and from mixing true positives from different calls. A modification that favors locations that include strong detections on receivers near the location only partially resolves this problem. For the Cascadia Initiative data set, the algorithm is more suitable for finding groups of locations that are consistent with calls from a single whale. It is inferred that such groups are reliable when they include at least 20 locations. The location uncertainties are quite large but methods to fit a smooth track to the locations in a group suggest that the net speed and swimming direction of the whale can be estimated when the path is ≥10 km long.

There are several ways that the localization method could be improved for future experiments. Efforts to improve the detection algorithm might reduce the number of false positives. Obviously if the receivers were more closely spaced, there would be less need to incorporate the weak low probability detections that lead to many spurious locations. Unfortunately, the needs of marine mammal studies are unlikely to influence the design of OBS experiments. A more realistic modification, given the ongoing improvements in storage capacity and power requirements of OBSs, is that future experiments sample at higher rates. For OBSs with the anti-alias filter set to ≥50 Hz (i.e., sample rates >100 Hz), a spectrogram cross-correlation detector could also search for harmonics of the B call [36]. The third harmonic in particular, has a high amplitude [37] and a sweeps down over a frequency band that is three times as large and it is thus, more diagnostic than the first harmonic. Using higher harmonics would also provide a means to eliminate spurious detections from the A call. The A call is not swept in frequency, but its frequency content overlaps that first harmonic of the B call [38] and thus, strong A calls often register as weak B call detections in our data set. If B calls are detected using the first harmonic, then A calls could be eliminated by using an automated A call detector but these are hard to implement because of the pulsed character of the call [36]. However, since A calls do not have significant energy that overlaps the third harmonic of the B call, they will not trigger a B call detector based on this harmonic. Finally, if detections are made on the third harmonic, the detection time uncertainty would likely be reduced to about a third because it is inversely proportional to the rate of frequency modulation. Smaller detection time uncertainties could be combined with more sophisticated travel time modeling, to obtain locations with much smaller travel time misfits. This would in turn, reduce the possibility that spurious or mismatched detections would randomly combine into spurious locations with travel time misfits comparable to true locations.

## Supporting information

S1 Dataset Call detections. The call detection data is in a ZIP file that includes a single comma separated variable file in which each row after the header row contains a call detection with the station name, station longitude in decimal degrees, station latitude in decimal degrees, numerical date in “mm/dd/yy” format, UTC time in “HH:MM:SS.SSS” format, the peak recognition score, the duration, and the estimated probability that the detection is a true positive.
(CSV)
